# Advanced Electric Discharge Machining of Stainless Steels: Assessment of the State of the Art, Gaps and Future Prospect

**DOI:** 10.3390/ma12060907

**Published:** 2019-03-19

**Authors:** Jaber E. Abu Qudeiri, Ahmad Saleh, Aiman Ziout, Abdel-Hamid I. Mourad, Mustufa Haider Abidi, Ahmed Elkaseer

**Affiliations:** 1 Mechanical Engineering Department, College of Engineering, United Arab Emirate University, Al Ain 15551, UAE; ziout@uaeu.ac.ae (A.Z.); ahmourad@uaeu.ac.ae (A.-H.I.M.); 2 Department of Mechanical Engineering, Zarqa University, Zarqa 13132, Jordan; abuhussein@zu.edu.jo; 3 Mechanical Design Department, Faculty of Engineering-El Mataria, Helwan University, Cairo 11795, Egypt (On leave); 4 Raytheon Chair for Systems Engineering (RCSE Chair), Advanced Manufacturing Institute, King Saud University, Riyadh 11421, Saudi Arabia; mabidi@ksu.edu.sa; 5 Institute for Automation and Applied Informatics, Karlsruhe Institute of Technology, 76344 Karlsruhe, Germany; ahmed.elkaseer@kit.edu; 6 Department of Production Engineering and Mechanical Design, Faculty of Engineering, Port Said University, Port Said 42523, Egypt

**Keywords:** EDM, stainless steels, machining, process parameters, processes responses

## Abstract

Electric discharge machining (EDM) is a material removal process that is especially useful for difficult-to-cut materials with complex shapes and is widely used in aerospace, automotive, surgical tools among other fields. EDM is one of the most efficient manufacturing processes and is used to achieve highly accurate production. It is a non-contact thermal energy process used to machine electrically conductive components irrespective of the material’s mechanical properties. Studies related to the EDM have shown that the process performance can be considerably improved by properly selecting the process material and operating parameters. This paper reviews research studies on the application of EDM to different grades of stainless steel materials and describes experimental and theoretical studies of EDM that have attempted to improve the process performance, by considering material removal rate, surface quality and tool wear rate, amongst others. In addition, this paper examines evaluation models and techniques used to determine the EDM process conditions. This review also presents a discussion on developments in EDM and outlines the likely trend for future research.

## 1. Introduction

In recent years, the rapidly rising demand for materials with special characteristics in such advanced industrial applications as aerospace and surgical instruments, has led to the development of new materials. However, these materials are mostly difficult-to-cut using more conventional manufacturing processes [1,2,3,4,5] and this pushes manufacturers to explore new machining processes which maintain or even improve precision but at reasonable cost.

Stainless steel is one of the widely used difficult-to-cut materials because of its superior properties which combine good corrosion and chemical reaction resistance, with the ability to be easily cleaned, polished and sterilized. New stainless steel compositions are developed to meet the need for higher corrosion resistance, increased strength and elevated temperature resistance. As mentioned in Reference [6], about 150 separate and distinct compositions of stainless steels already exist. These compositions include grades #304, #305 and #316, each of which was developed to meet a specific end-use and each of which—in common with most stainless steels—contain common alloying ingredients, such as chromium, nickel or molybdenum [7].

Electric discharge machining (EDM) is one of the most advanced and successful manufacturing methods used to machine materials that are difficult-to-cut [8,9,10,11,12]. EDM is being used in modern industries to facilitate complex machining processes and achieve highly accurate machining [13,14,15,16,17,18,19,20]. EDM is utilized to remove material from a conductive workpiece by repetitively applying sparks between the EDM electrode tool or wire and the workpiece. In this process, no mechanical cutting forces are applied because no contact exists between the electrode tool and the workpiece [17,21,22,23,24]. The fundamental principles of the EDM process are applied in many processes, including: die-sinking EDM, wire EDM, micro-EDM, powder-mixed EDM and dry EDM. These variants make the process suitable for machining components from the relatively large to the micro-scale.

The EDM process has advantages over other machining processes. EDM can machine complex shapes and extremely hard materials as described in a number of publications [9,25,26,27,28,29,30]. The EDM can be used to machine very small, delicate and fragile products without damage because no cutting forces are applied and hence there are no mechanical induced residual stresses. However, EDM has its own limitations with regards to the workpiece material and shape [26,31,32]. At present EDM can only be applied on electrically conductive materials. The process has low material removal rate and high electrical power consumption. Furthermore, additional cost is incurred preparing the electrode tool in case of the die-sinking EDM. Finally, sharp corners are difficult to produce using EDM because of electrode tool wear.

While many studies have reviewed EDM, wire EDM and other EDM processes [10,33,34,35,36,37,38,39], no study has reviewed and reported on the use of EDM for machining of stainless steel specifically, though there are many reviews available on other materials machined by EDM.

**Figure 1:**
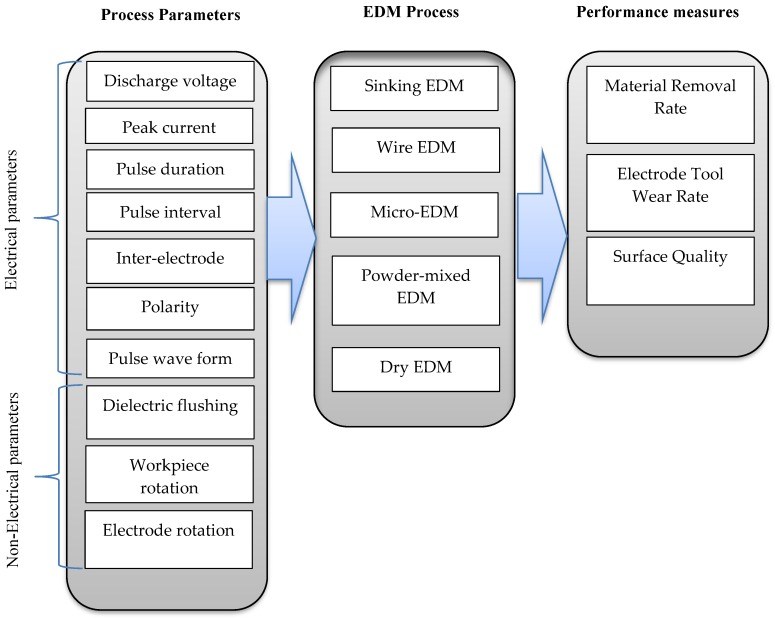
Electric discharge machining (EDM) processes, process parameters and performance measures.

This study aims to provide an overview of the significant contributions of EDM to the machining of various stainless steel variants. This paper reviews the research studies that used different EDM variants for machining different types of steel materials. The paper starts with a brief introduction of EDM and its development, then it provides the working principles of this machining method. EDM process parameters and performance measures are then discussed. Next, the paper presents the various types of EDM processes. This study also provides a review of the major areas of research into the application of EDM to different grades of steel. The conclusions drawn by and the trend of, the reviewed research are presented and discussed. Figure 1 shows the EDM processes and their main input (process) parameters and output (performance) measures.

## 2. General View of the EDM Method

### 2.1. EDM Principles

**Figure 2:**
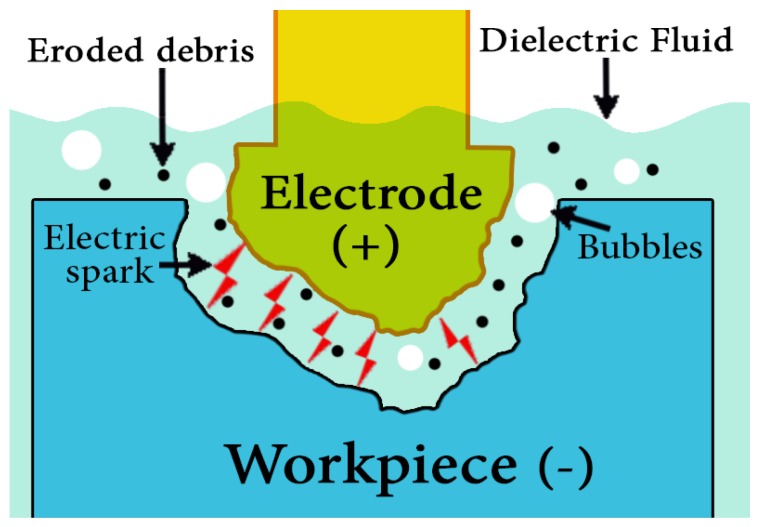
Principle of EDM.

The EDM manufacturing process was invented in the 1940’s [17]. The principle of the EDM technique is to use thermoelectric energy to erode a workpiece by automatic spark repetition [40,41,42,43]. The rapidly recurring electrical discharges (sparks) between a non-contact electrode tool and the workpiece allow erosion caused by sparks generated between electrode tool and the workpiece surface [44]. In this process, both the workpiece and the electrode tool are submerged in an insulating dielectric fluid. The gap between the electrode tool and the workpiece is carefully selected so that the voltage across the gap has a value that can ionize the dielectric fluid in the gap due to electrical breakdown. Discrete electric discharges between the electrode tool and workpiece are produced which in turn generates a high temperature plasma channel, where instant thermal dissipation occurs. The local high temperature melts both workpiece and tool. Then, the eroded material solidifies in the form of debris. Flushing the dielectric fluid during the machining process carries away debris (separated solid particles) and restores the sparking condition in the gap and avoids short circuiting. No cutting forces exist between the electrode tool and the workpiece because there is no contact between them. This minimizes the vibration and stress problems that can occur during machining [45,46,47,48]. A principle of EDM is shown in Figure 2.

### 2.2. EDM Process Parameters

The EDM process is driven by both electrical and non-electrical parameters. The major electrical parameters are discharge voltage, peak current, pulse duration and interval, electrode tool gap, polarity and pulse waveform. The non-electrical parameters include rotation of the electrode tool, the flushing action of the dielectric fluid and the properties of the workpiece. These parameters are described in this section.

The discharge or machining voltage is the average voltage in the spark gap during machining. The electrical potential drops sharply after the open gap voltage because of the discharge and the current flow rises. The machining will begin at the working voltage. The discharge voltage directly influences the size of spark gap and overcut [49,50,51,52,53,54]. A low voltage is normally used with electrode tool and workpiece materials that possess high electrical conductivity. In contrast, materials with low conductivity use a much higher voltage [55]. The peak current, which is defined by the maximum power spent in discharge machining, is a parameter that highly influences the EDM process. The peak current is represented by the maximum level that is reached during the on-time of each pulse. This parameter has a direct effect on the material removal rate (MRR), tool wear rate (TWR) and machining accuracy [56,57,58,59,60,61]. These characteristics make it very important and has resulted in research into high wear resistance that can occur with high current conditions [47].

The pulse on-time is the duration for which the discharge is applied. A high-temperature plasma channel heats both the electrode tool and the workpiece during the discharge. The amount of energy generated during the pulse on-time has a direct effect on the MRR [62,63,64,65,66,67]. Increasing the discharge energy by applying longer pulse on-times increases the MRR [68,69]. Debris form during the discharging period, creating an insulation layer and lead to arcing. This layer can be flushed away during pulse-off time. The pulse off-time is the time in which no discharge is applied. Proper selection of the pulse off-time provides stable machining [22,25,36,70,71]. A shorter period can increase the machining speed but off-time should be long enough to allow flushing away of debris from the gap; otherwise, it may result in unsuitable conditions for the next on-time pulse [72], taking into account that long breaks between pulses can cause overcooling the machined material which has impact on MRR. The pulse wave form is usually rectangular in shape, to reduce electrode tool wear other pulse shapes have been used, for example, trapezoidal [73,74]. Another generator has recently been developed to facilitate initiation of the main pulse by producing a high voltage pulse with a low current for a short period before the main pulse [22].

The effect of the EDM process parameters on performance cannot be easily explained because of the stochastic nature of the discharge mechanism [75]. Thus, many studies related to EDM have explored the influence of the process parameters on performance measures and have introduced the concept of optimal process parameters that achieve best performance [18,63,64,76,77,78,79].

The electrode tool polarity in the EDM process can be positive or negative and this determines the direction of the electrical current, from or toward the electrode tool. The choice of polarity depends on many factors, including electrode tool and workpiece materials, current density and pulse length [80,81,82]. In die-sinking EDM, the generators have the flexibility to switch to either a positive or a negative electrode tool polarity based on the machining requirements. Positive electrode tool polarity is generally used in EDM operations because electrode tool wear will be lower. The negative electrode tool polarity is a better choice if a high MRR is more important than precision. Nevertheless, this is at the cost of very high electrode tool wear. Negative electrode tool polarity machining conditions are suitable for machining materials, such as carbide, titanium and copper alloys, amongst others. In the wire-EDM process, the electrode “wire” usually has a negative polarity because a high machining rate is required and the wire wear is not important because the wire can be fed continuously to replace the eroded portion.

The necessary sparks do not occur if the electrode tool and the workpiece touch each other. Thus, the electrode tool and the workpiece are separated by a small distance called the “inter-electrode gap.” The discharge gap is controlled by the discharge gap servo that maintains the proper separation [83,84] which is normally between 0.005 mm and 0.1 mm. The electrode tool is moved up and down during machining to enable proper evacuation of the debris. The discharge occurs during the down period and the up period allows the flushing of the debris away from the machining area. For finishing and micro EDM processes, RC generator is usually used. The RC pulse generator is a low-cost power source for EDM and principally a relaxation oscillator with a resistor and a capacitor. It can produce very small pulse energy that generates small craters which in turn lead to produce small surface roughness. However, lack of precision control is the main disadvantage of RC generator especially for timing and slow charging [10,13,85,86,87].

The main non-electrical parameters are the flushing of the dielectric fluid, workpiece and electrode tool rotation. The EDM process needs a dielectric fluid medium that submerges both the electrode tool and the workpiece to at least a suitable distance above the gap between them. In addition to high dielectric strength, the dielectric fluid must have a flushing ability and fast recovery after breakdown. The dielectric fluid provides insulation against premature discharging, reduces the temperature in and around the machined area and cleans away the separated debris.

For the die-sinking EDM, the dielectric fluid is a hydrocarbon and silicone-based dielectric oil and kerosene with an increased flash-point. Some die-sinking EDMs use de-ionized water for high-precision machining, such as fine hole drilling. De-ionized water and oil are also used with wire EDM. Many studies have recently been conducted to explore the use of oil-based synthetics to avoid harmful effects to the worker and the environment [48,88,89]. Previous studies have reported that the dielectric type, flushing method and flushing pressure influence the MRR, TWR, surface roughness (SR) and surface quality (SQ) [90,91,92,93]. Dielectric flushing is improved with workpiece and electrode tool rotation [94,95]. The improvement in flushing due to electrode tool rotation achieves a better SR and a higher MRR [96,97,98]. Selecting the optimal flushing pressure can minimize the density of the crack and the recast layer [92].

### 2.3. Performance Measure Parameters

The performance parameters are the factors that measure the performance of the EDM process. These parameters include the MRR, TWR and SQ. The MRR is a measure of the performance of the erosion rate of the workpiece surface and an indication of the machining ratio. The MRR is usually expressed as the volume of the removed material per unit time. Techniques and methods to improve the MRR have attracted attention because the MRR represents the machining speed [29,99,100,101,102,103]. The TWR is a measure of the erosion rate of the electrode tool and has a direct influence on the shape of the machined cavity because of the continual change in the electrode tool profile during the machining process. Similar to the MRR, the TWR can be expressed by the volume of material removed per unit time. Previous studies focused on reducing the TWR because the wear of the electrode tool affects the electrode tool profile and leads to a lower precision [78,104,105]. The SQ is a measure of the quality of the machined surface and includes many components, such as the SR, extent of the heat affected zone (HAZ), recast layer thickness and micro-crack density. Many research studies have explored utilization of the EDM process in surface treatment and have reported the SQ generated by the process [106].

### 2.4. Types of EDM Processes

#### 2.4.1. Die-Sinking EDM

In the die-sinking EDM process, the workpiece is machined by a controlled electrical spark generated in the gap between the electrode tool and the workpiece. Sparking is repeated until the electrode tool shape is replicated in the workpiece surface facing the electrode tool. The heat produced by the electrical spark causes a sharp temperature rise in the area to be machined (i.e., 8000 to 12,000 °C). EDM machines contain a unit that controls and monitors the machining variables, such as the gap and axis movements. Furthermore, this system shows the process execution sequence.

**Figure 3:**
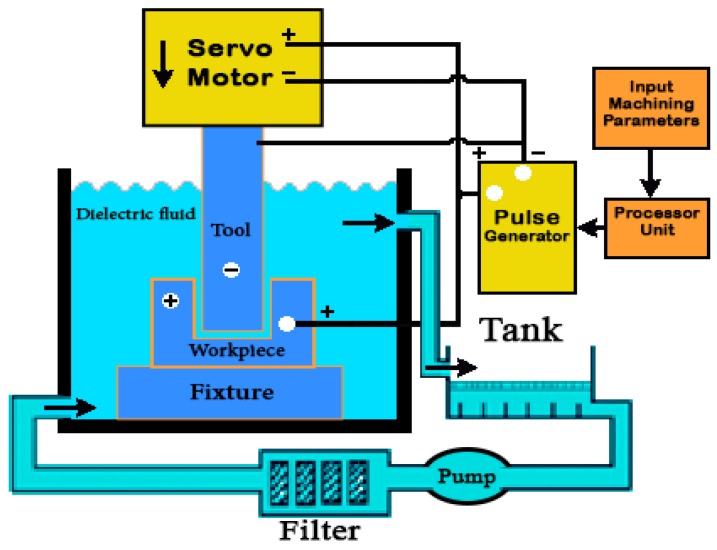
Schematic diagram of the die-sinking EDM.

Normally, copper or graphite is used as the electrode tool material in this process with hydrocarbon dielectric because of its positive effect on the SR and EWR (Electrode tool Wear Rate). The dielectric flows through the cooling system, carrying the debris and eroded material with it, is filtered to remove the suspended particles and is returned to the system. In the die-sinking EDM process, the electrode should be re-shaped to carry out the finishing operations. Figure 3 shows a schematic diagram of the die-sinking EDM.

#### 2.4.2. Wire EDM

**Figure 4:**
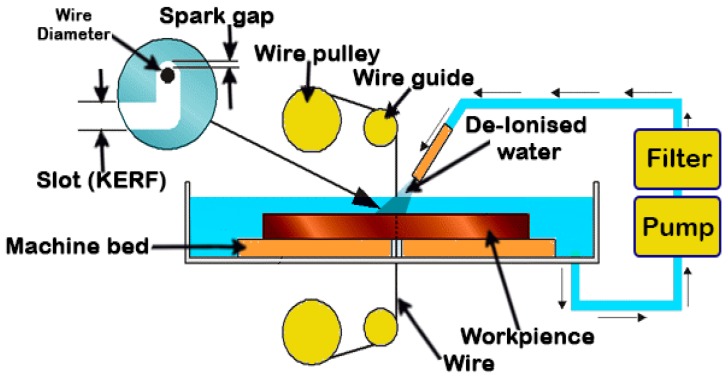
Schematic diagram of the wire EDM.

In the wire EDM, a metallic thin wire is used to cut the workpiece along a well-defined path. Discrete sparks between the wire and the workpiece cause eroding in the machined area. The wire used is usually thin, the standard EDM wire is 0.25 mm. Micro-wires dimeter can range from 0.020 mm to 0.15 mm [107] and is normally copper, brass or coated steel materials. As with the die-sinking EDM, the wire and the workpiece do not have any contact during machining [108] and both should be immersed in a dielectric fluid. A high peak current of short duration is applied in this process. The machining variables and the movement of the worktable that holds the workpiece are controlled by the control units. Thus, complicated shapes can be produced using this process [9,14,27,109,110,111,112,113,114]. The control unit contains a microprocessor to maintain the gap between the wire and the workpiece in a suitable range, normally between 25 µm and 50 µm. In addition, the unit controls the feeding of the wire through the workpiece at a suitable speed that produces surfaces with very high accuracy. De-ionized water is a common dielectric fluid used in this process. The wire EDM process has a wide range of applications, such as in die making, electronics and automotive industries [115,116]. Closed operations, which do not start from the edges of the workpiece, require the drilling of a full-depth hole to start the machining process. Figure 4 shows a schematic diagram of the wire EDM.

#### 2.4.3. Micro EDM

The micro-EDM is a machining process that follows the same principles as the die-sinking EDM and wire EDM. The process removes material at the micro-scale for components smaller than 100 μm, including micro-holes, micro-shafts and 3D micro-cavities [117]. The only principled difference from the other EDM processes is the power involved [51,118,119,120]. The MRR will be in nanometres because the machine part is at the micro level and the required voltage and current will be several times less than those used in the die-sinking EDM or any other normal-level EDM process. This process can produce a hole or shaft diameters of only 5 µm, while holes of up to 70 µm and 40 µm can be produced by drilling and laser machining, respectively [121]. EDM at the micro-scale level is available in many machining processes, such as die-sinking micro-EDM [122], micro-wire EDM [123,124], micro-EDM drilling [125,126,127,128] and micro-EDM milling [129]. In the micro-wire EDM, a wire with a diameter less than 20 μm is used. The minimum machinability of cavities in other micro-EDM processes have diameters of 5 µm. The grain sizes of workpiece materials have significant impact on the characteristics of micro EDM [130]. The MRR for micro-EDM has a direct relationship with the grain size of the machined workpiece because the effective thermal conductivity and local effective melting point of polycrystalline materials vary with grain sizes of these materials since the grain boundary volume fractions change [131]. It is worth emphasizing that the material microstructure of the processed workpiece plays a significant role in the performance of the micro EDM process. In particular, the refined material microstructure can give a better surface quality when compared with the results for the course grained microstructure of the same material. This conclusion is explained by the heterogeneity of the course grained material microstructure that normally leads to more anisotropic behaviour of the microstructure and the more homogenous response of the refined microstructure that results in more isotropic/consistence behaviour [132].

#### 2.4.4. Powder-Mixed EDM

As the name implies, powder of a suitable material, such as nickel, is mixed with the dielectric fluid. The presence of this powder makes the process mechanism substantially different from the conventional EDM process [133,134]. The powder particles fill the gap between the electrode tool and the workpiece when a voltage is applied during machining. This particle aggregation forces the electrode tool and the workpiece to move a small distance further apart by an amount equal to the gap filled by the powder particles. The gap between the electrode tool and the workpiece can increase by 100% to 300% (from 25–50 μm to 50–150 μm) [135]. The presence of the powder particles between the electrode tool and the workpiece leads to earlier and faster sparking, which causes a higher erosion rate.

#### 2.4.5. Dry EDM

The dry EDM uses dielectric high-pressure gas instead of dielectric liquid [12,136,137,138,139]. Here, the electrode tool is in the form of a thin-walled pipe through which high-pressure gas or air is supplied [140]. The pressurized gases flow outwards through the gap between electrode tool and machined surface and carry away the debris being formed. The gases also reduce the machining area temperature. Using gas instead of fluid in this process can reduce harmful environmental effects. Most notably the dielectric fluid and the powder-mixed dielectrics in the EDM processes are associated with evaporation from the fluid surface during machining. Utilizing gas can also decrease the cost of managing the debris waste and enhance the machining performance and the environment as regards worker health. From this point of view, this process could be named as the “Green EDM.” The dry EDM process positively influences the MRR [138,141] and reduces the EWR [142]. Under ideal conditions, this process allows to obtain very good accuracy and surface layer quality [143].

In addition to the previous main types of EDM, there are other types such as EDM milling, in this type the final shape is obtained using a simple electrode tool which is moved in a 3D path along several directions and may also subject to rotations [144]. A combination of the two cutting systems can also be applied. Also, EDM grinding, when the electrode tool design as a rotating disk [145].

### 2.5. Mathematical Modeling of the Thermos-Physical Phenomenon in EDM

EDM involves removal of material from the workpiece due to heat generated from electric discharge in the inter-electrode gap. Plethora of research study and analyse this phenomenon; mathematical models are developed to provide better understanding of the EDM process. A quasi-static model is proposed by [146], the model computes the material removal rate based on predicted distribution of the temperature in the workpiece. Equation of transient heat conduction is employed to predict the distribution. The model assumes Gaussian heat flux since it gives better results as demonstrated by [147]. Finite element method is used to solve the model and obtain the results, which show significant closeness to the experimental results. Vaporization of workpiece and tool materials is studied by [148]. The model is used to predict the aerosol emission of EDM process. 70% of the aerosol is found to be vaporized material from the workpiece and the tool, the rest comes from the dielectric fluid.

The electric field generated in the interelectrode gap is modelled by [149]. The model represents the electric field at two stages. First; before-discharge stage, where Laplace equation is used to model the electrostatic field. Second; during–discharge state, where Poisson equation is used to model the spatial discharge from electrode and particles of the dielectric fluid. Fluid flow in the interelectrode gap is modelled by [150]. The model attempts to study the motion of the debris particle as well as the drag force between the particles and the dielectric fluid. The purpose is to improve the removal of debris from the machining zone. Fluent software is used to build 3D model of drilling high aspect ratio of a hole. The effect of incorporating ultrasonic vibration is verified using the proposed model; optimal amplitudes and frequencies are determined using the model based on a set of process parameters.

## 3. Different Stainless Steel Grades

Steels can be categorized into four groups; stainless steel, tool steel, carbon steel and alloy steel. Each of these groups has its own characteristics, which makes it suitable for specific applications. This paper focuses on stainless steel. This group has good corrosion and chemical reaction resistance. Stainless steel can be divided into three classes; martensitic, ferritic and austenitic. All stainless steels contain common alloying ingredients, such as chromium (minimum of 11%), nickel and molybdenum [7]. The composition and properties of some stainless steels are given in the Table 1. As mentioned in Reference [7], there are actually about 150 separate and distinct compositions and each of them serves specific requirements. Stainless steel is widely used in household cutlery, food handling/processing, hardware, surgical instruments and structural/architectural applications [151,152,153].

**Figure 5:**
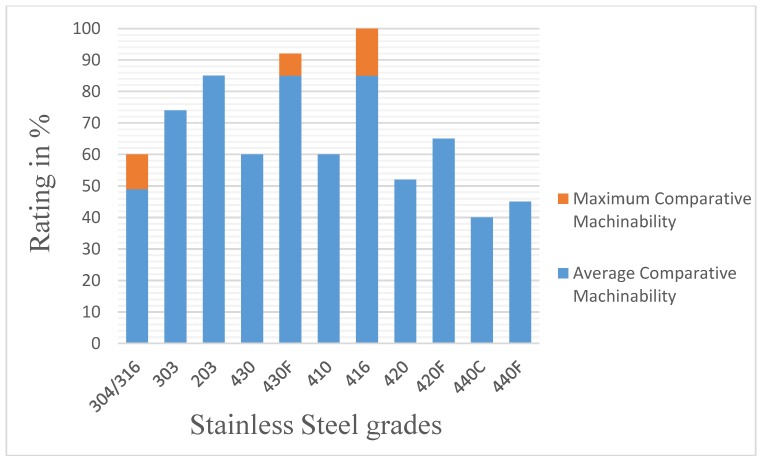
Comparative machinability of frequently used stainless steels and their free-machining counterparts.

The machinability, which is defined as the speed at which a material can be cut [154], is different for the different stainless steel grades. The 400 series is most easily machined, whereas the 200 and 300 series are the most difficult [155]. Figure 5 presents the comparative machinability of the most frequently used stainless steel.

## 4. Research on EDM of Stainless Steel

This section will discuss research that considered the performance of different grades of stainless steels subject to EDM processes. Table 2 shows a summary of the studies.

### 4.1. Performance Measures for the EDM of Stainless Steel

**Figure 6:**
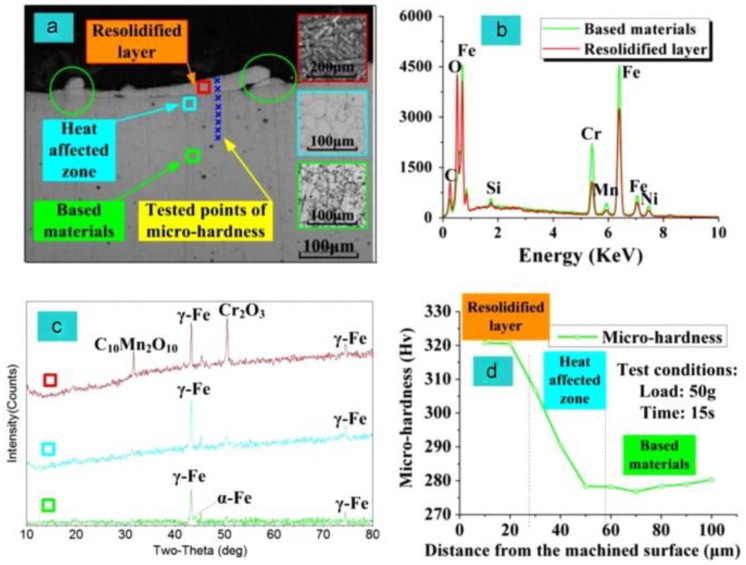
(**a**) Scanning Capacitance Microscopy (SCM) photograph of the cross-section of the AISI304 in high-speed dry EDM; (**b**) EDS spectra of the re-solidified layer and base materials; (**c**) XRD diffractograms of the re-solidified layer, heat affected zone and base materials; and (**d**) micro-hardness of the cross-section [48].

A significant amount of research has been conducted into the effect of working parameters on the processing performance of EDM of stainless steel [74,77,181,204,206,210,211]. Many methods have been introduced to improve performance. Shen, et al. [48] recently proposed high-speed dry EDM to improve MRR. Utilizing the proposed method, the material rapidly melted by the high-discharge energy and was flushed out at high pressure. The authors considered the influence of workpiece polarity, discharge current, pulse duration, gas pressure and electrode tool rotation speed on the machining performance. Moreover, the study investigated the solidified layer, surface morphology, composition of the working material and phase of the AISI 304 stainless steel used with the high-speed dry EDM. Figure 6 shows some of the results from this paper.

**Figure 7:**
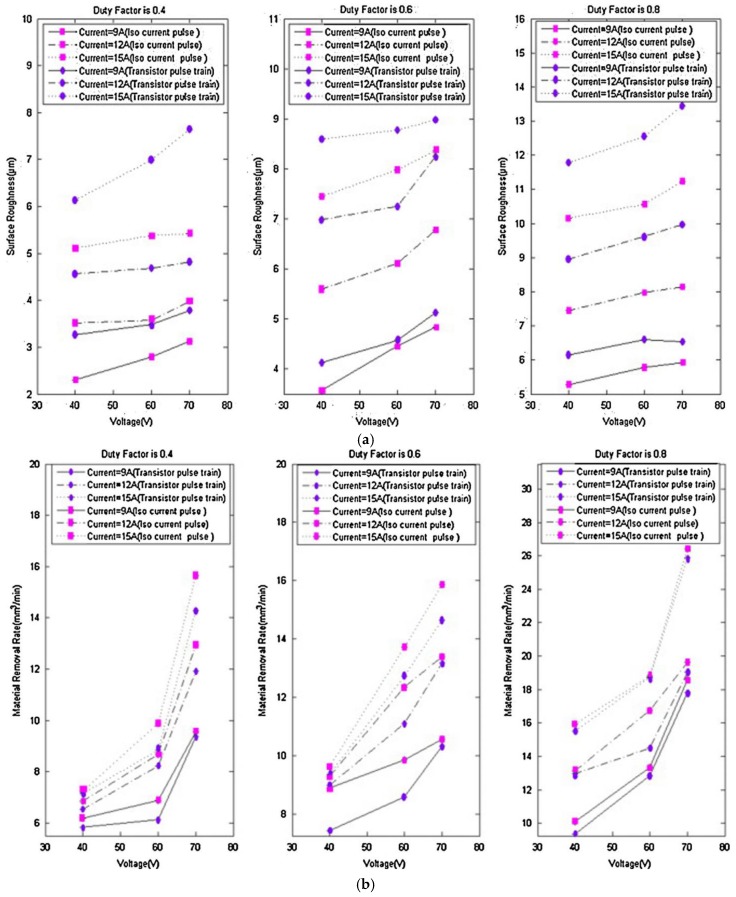
(**a**) Effect of pulse generators on the material removal rate; and (**b**) effect of pulse generators on SR [188].

Ugrasen, et al. [193] used multiple regression analysis and the group method data handling technique to develop a model for predicting the parameters that defined machine performance. The effects of the cutting parameters, including pulse-on, pulse-off, current and bed speed with constant voltage and flush rate on the four response parameters (accuracy, SR, volumetric MRR and TWR), were discussed. Muthuramalingam and Mohan [188] studied the surface finish obtained using an iso duration current pulse generator. The authors reported that, the modified iso duration current pulse generator produced a better SQ with a higher MRR than the conventional transistor pulse train generator. Figure 7 shows the effect of pulse generators on the material removal rate and on SR.

Castillo, et al. [164] studied the effect of the processes parameters including pulse current, pulse on time and pulse off time on the surface roughness of AISI 304 stainless steel workpieces. The authors reported that the pulse current and pulse on time are the most significant machining parameters on the obtained surface roughness values of the workpieces machined by EDM. Also, the study presented regression analysis of a second order model to estimate the average roughness in terms of the pulse current, pulse on time and pulse off time. Roth, et al. [177] studied the MRR in different gaseous fluid environments with molecular oxygen gas. They measured the voltage and current values in the gap and used these to calculate the effective energy specific values of the MRR. These authors reported that increasing the oxygen gas of the fluid environment increases the MRR for single sparks and the process time efficiency. The micro-hole operation was discussed by Li, et al. [205], their paper studied the SQ of the micro-hole with different power supply modes.

**Figure 8:**
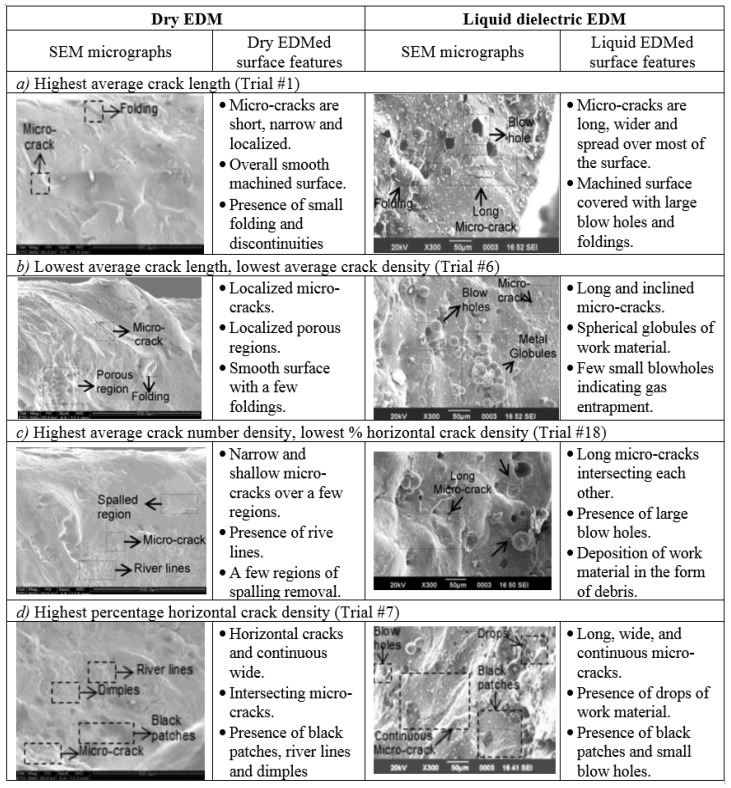
A comparison of the machined surface topography in dry and liquid EDM [175].

Allen and Lecheheb [212] developed an understanding of the effects of the Micro EDM on the hole properties. Yahagi, et al. [160] investigated the effect of high spindle speeds on the machining performance. Their study reported on the effect of high spindle speed on the micro drilling of deeper holes with a lower tool wear ratio, when machining stainless steel (SUS304) plate. Son, et al. [158] investigated the influence of the EDM pulse condition, in particular pulse duration and the ratio of on-time to off-time, on the machining properties; TWR, MRR and machining quality. Govindan and Joshi [175] studied micro-crack formation, discussing the influence of the machining parameters on crack formation, micro-crack length, orientation and number of cracks. The paper also compared the crack formation of dry EDM with liquid dielectric EDM. The results showed that the average length and number density of the micro-cracks were lower with dry EDM than with liquid dielectric EDM (Figure 8).

**Figure 9:**
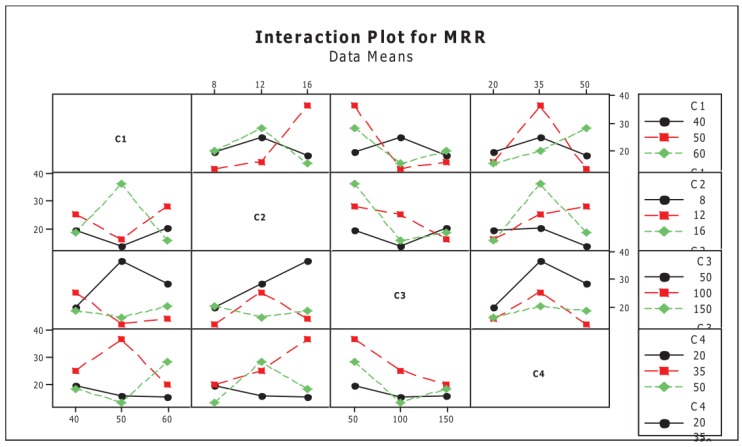
Interaction plot of the material removal rate (MRR) [157].

Jegan, et al. [56] studied the effect of the machining parameters, including discharge current, pulse on-time and pulse off-time on the performance of EDM when machining AISI 202 stainless steel. They utilized the grey relational analysis to find the optimal performance parameters such as MRR and SR. Rajmohan, et al. [157] studied the effect of EDM machining parameters such as pulse-on time, pulse-off time, voltage and current on MRR for 304 stainless steel signal to noise ratio (S/N) and analysis of variance (ANOVA) was used. Experiments were carried out as per design of experiments approach Taguchi table orthogonal array to analyse the effect of process parameters on MRR and also to identify the optimum cutting parameters, the interaction plot of MRR is shown in Figure 9.

**Figure 10:**
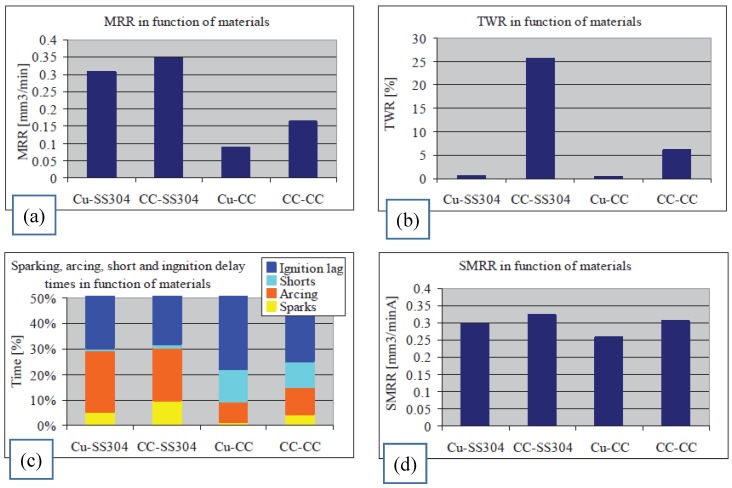
(**a**) MRR dependence on anode and cathode material combinations; (**b**) TWR as a function of anode and cathode material combinations; (**c**) sparking, arcing, shorts and ignition delay times as a function of anode and cathode material combinations; and (**d**) current specific MRR as a function of the anode and cathode material combinations [176].

Roth, et al. [176] studied the MRR for different electrode tool and workpiece materials in terms of the breakdown behaviour of the process. The authors reported that the breakdown mechanism was different from the traditional EDM when the work gap was filled with gas rather than liquid dielectric. They presented MRR, TWR and specific MRR, as well as the sparking, arcing and ignition delay times on the function of the anode material (see Figure 10).

**Figure 11:**
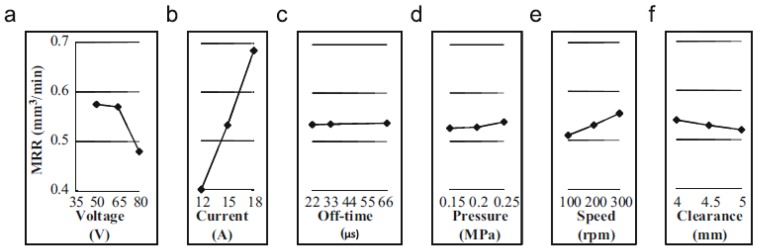
(**a**–**f**) Main effects of the input parameters associated with the MRR [174].

**Figure 12:**
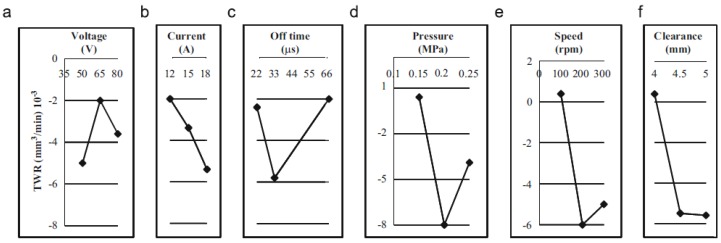
(**a**–**f**) Main effects of input parameters associated with the tool wear rate (TWR) [174].

Govindan, et al. [174] presented an experimental characterization of 304 stainless steel removal using the dry electrical discharge drilling technique. They selected various independent parameters for the experiments. All the experiments were performed in a ‘quasi-explosion’ mode by controlling the pulse off-time. The main response variables analysed in this work were MRR, TWR, over size and compositional variation across the machined cross-sections. The authors reported that the discharge current, gap voltage and rotational speed significantly influence the MRR. The TWR was found close to zero in most of the experiments. The paper presented the influence of the machining parameters in the MRR and TWR (Figure 11 and Figure 12).

**Figure 13:**
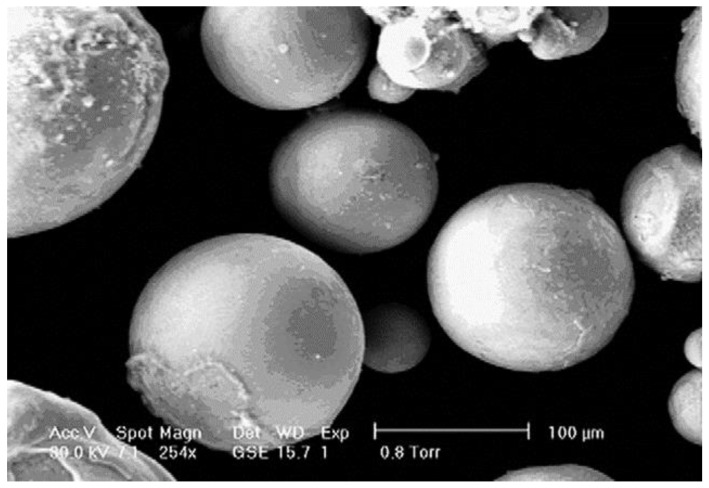
Scanning electron micrograph of particles produced in water at 50 A and 512 ms [170].

**Figure 14:**
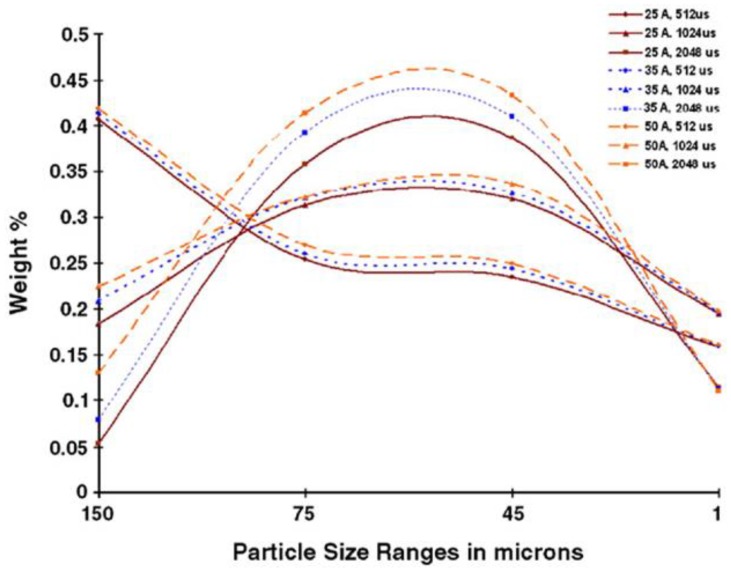
Particle size distributions in deionized water with varying current intensities and pulse widths [170].

Vasudevamurthy and Knight [170] studied the effect of the process parameters on the size distribution of the 304 stainless steel particles produced by the electrical discharge mechanism. They obtained empirical data on the process parameters for the uranium carbide microsphere modelling as shown in Figure 13 and Figure 14. Figure 13 shows a scanning electron micrograph of the micro-particles produced in water with an arc of magnitude 50A and a pulse width of 512 μs.

Figure 14 shows the particle size distribution with varying current intensities and pulse widths when deionized water was the medium.

Rebelo, et al. [213] studied the effect of the EDM parameters on SR, metallurgical structure, residual stress state and surface crack network in martensitic steels that underwent EDM. They qualitatively and quantitatively examined and assessed these effects. Artificial neural networks and response surface methodology were utilized in many studies to model the EDM processes. Tang and Guo [211] combined the Deng Grey Incidence Analysis model and Taguchi orthogonal array analysis of the experimental results to optimize electrical discharge machining parameters with a high level of accuracy. The results showed that the obtained optimized parameters increased the MRR by almost 24%.

Spedding and Wang [192] used two techniques to develop models for the wire EDM process: the response surface methodology and an artificial neural networks. They investigated SR, skewness, kurtosis and waviness of a wire surface that underwent EDM. A measure of the surface waviness (as an output parameter) was included in the process modelling. Tarng, et al. [214] utilized a feed forward neural network to predict the influence of the cutting parameters on the cutting performance. They applied a simulated annealing algorithm to the neural network to find the optimal cutting parameters based on a performance index within the allowable working conditions. Based on the experimental results for SUS304 stainless steel it was claimed that the cutting performance of a wire-EDM can be greatly enhanced using this approach. Abdulkareem, et al. [215] investigated the effect of cryogenic cooling on reducing EWR in the EDM process. It was found out that with the help of cryogenic cooling the EWR was reduced by 27% and surface roughness improved. Srivastava and Pandey [216] also used cryogenic cooling of the electrode tool and showed that EWR was reduced by 20% and that SR was also reduced.

Jithin, et al. [184] studied the effect of operating parameters such as gap voltage and pulse on-time on the surface roughness of Stainless steel 316L and electrode tool of copper, tungsten and copper–tungsten were used. The authors reported that, at low level of operating parameters, the surface irregularities such as micro-globules and micro-cracks by copper electrode tool is lesser than the surface irregularities by other electrode tool materials. At high levels of operating parameters, a denser distribution of surface irregularities due to high electrical discharge efficiency was observed. Deris, et al. [185] conducted experimental study to investigate the influence of EDM parameters including peak current, servo voltage, pulse on time, pulse off time and servo speed on electrode tool wear rate value by using Grey Relational Analysis. The authors reported that peak current is the most significant parameter to the EWR value.

Boban, et al. [172] presented an experimental investigation to find the effect of polarity in tool wear micro EDM drilling of stainless steel SS 304. Three electrode tools were used namely, coper, brass and tungsten. The authors reported that, the direct polarity has significant influence in reducing the tool wear over the reverse polarity for the three electrode tools and the material removal rate is maximized with the direct polarity. Chandramouli and Eswaraiah [199,200] presented an experimental investigation to find the optimal process parameters of EDM including peak current, pulse on time, pulse off time and tool lift time on Material Removal Rate and Surface Roughness. They used L27 Taguchi experimental design to conduct the experiments on 17-4 Precipitation Hardening Stainless Steel (PH Steel). The authors reported that, peak current, pulse on time and tool lift time have significantly affected the material removal rate and surface roughness.

Ramachandra [180] optimized the machining parameters including Discharge current, Pulse on time and Duty cycle to maximize Material Removal Rate and minimize and Surface Roughness. AISI 316 stainless steel materials workpiece and Copper electrode tool were used. Taguchi’s L9 orthogonal array was used to study the response of control factors. Buschaiaha, et al. [162] presented an experimental study to characterize the electric discharge machining of AISI304 steel on EDM with the copper electrode tool. EDM parameters including as peak current, pulse duration and electrode tool diameter were considered to analyse the effect of each parameter on the machining characteristics. The authors reported that, these parameters have a significant influence on machining characteristic such that surface roughness. Table 3 summarizes the latest studies on optimizing the process parameters of the EDM machining.

### 4.2. Electrode Tool Shape and Movement Research in the EDM of Stainless Steel

**Figure 15:**
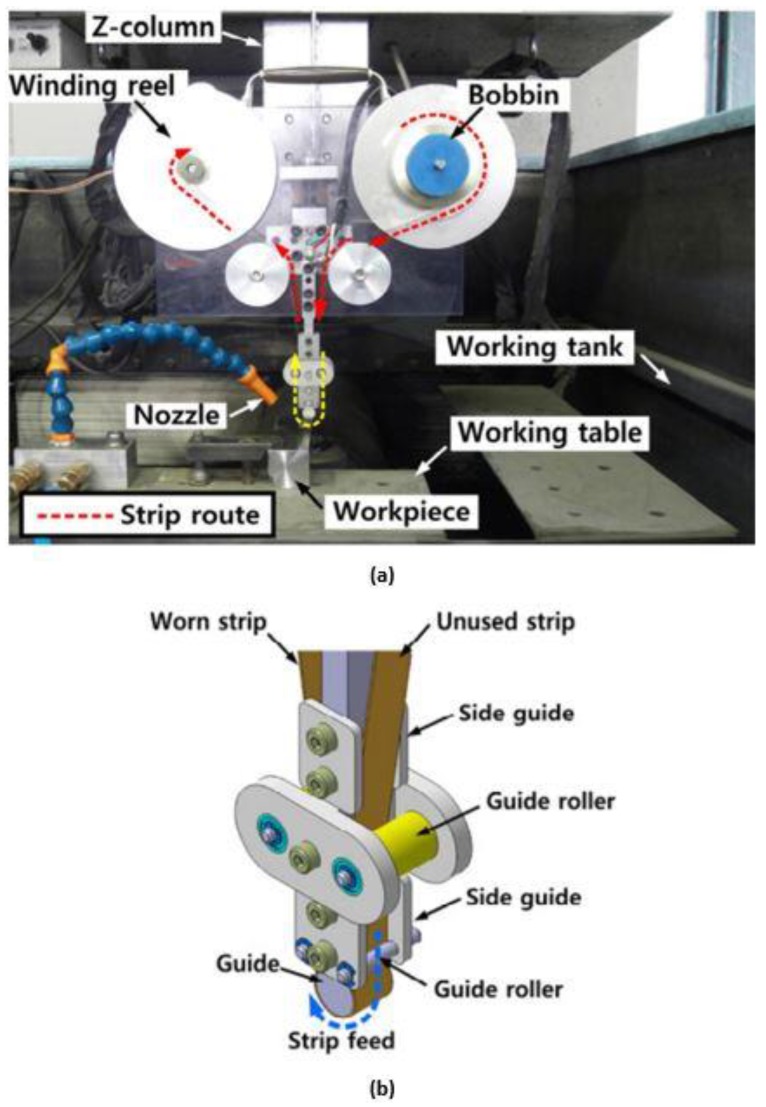
(**a**) Strip-EDM system; and (**b**) electrode guide [159].

**Figure 16:**
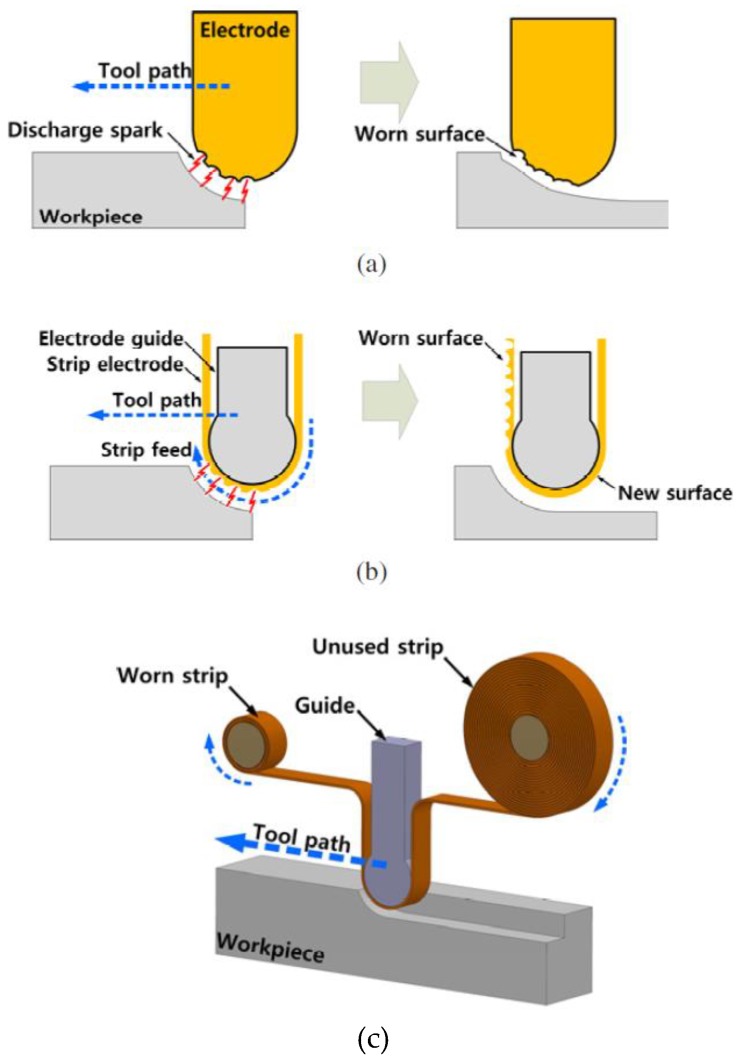
(**a**) General EDM; (**b**) strip EDM; and (**c**) concept of the strip EDM [159].

Stainless steel material has been considered in strip electrode studies [159,166]. For example, Song, et al. [159] proposed a strip electrode and guide system to minimize electrode tool wear during EDM. This was achieved by making the electrode a conductive strip which was continuously fed from a bobbin to a winding reel thus reducing electrode tool wear at any point on the strip, similar to that in the wire EDM. The strip EDM method was combined with EDM milling and wire-EDM and in addition to greatly reducing electrode tool wear, this method did not require the finish-cut process. The study applied the proposed method to the milling and turning of various stainless steel workpieces. Figure 15 shows the design of the apparatus using the strip electrode. The electrode tool wear when using conventional EDM was different from that obtained using a strip electrode because the latter was being continuously renewed with the eroded part of the strip replaced by a non-eroded portion. Figure 16 shows the wears in the conventional and strip EDM processes. Song, et al. [166] studied the usage of strip-EDM in the EDM-turning process as a means of overcoming tool electrode tool wear.

**Figure 17:**
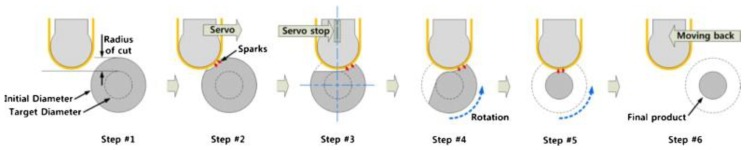
Strip-EDM turning process [166].

**Figure 18:**
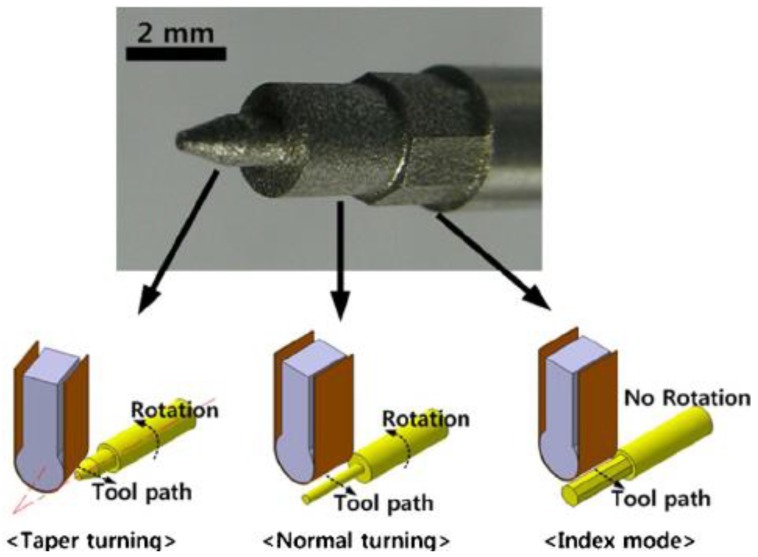
Product created using three kinds of strip-EDM turning [166].

Figure 17 shows the sequential steps of EDM with the strip electrode. Song, et al. reported that an advantage of strip-EDM, was that it increased the MRR because it has a larger machining area than a wire or non-breaking electrode. The study reported many experiments to determine the machining characteristics of strip-EDM using stainless steel 304 and complex shapes. Figure 18 shows the machined product using three kinds of strip-EDM turning.

**Figure 19:**
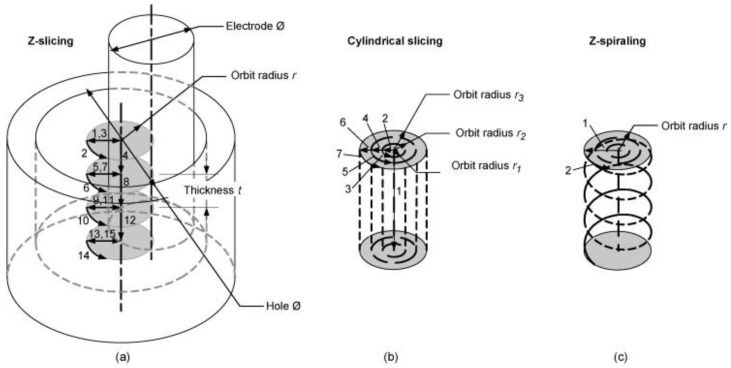
Electrode orbiting strategies: (**a**) slicing the hole into cylinders of thickness *t* and radius *r*; (**b**) slicing the hole into cylindrical shells of radius *r*; and (**c**) spiralling into the hole with radius *r* [182].

**Figure 20:**
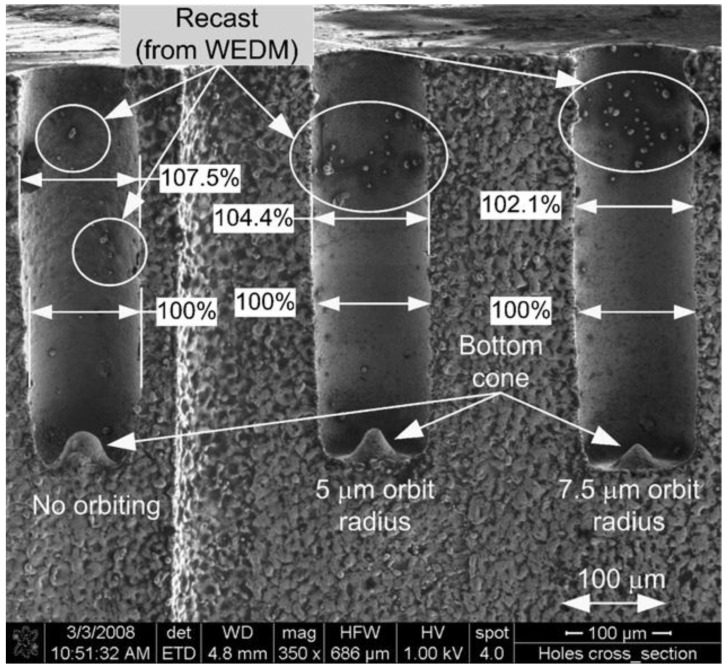
Cross-section of 500 µm deep micro-holes without orbiting; with 5 µm orbit radius; and with 7.5 µm orbit radius drilled with 60 V and 47 pF capacitance [182].

Other research has studied electrode tool movement. Bamberg and Heamawatanachai [182] presented a novel machining technique for the micro-EDM electrode tool orbiting of micro-holes. Figure 19 shows the electrode-orbiting strategies proposed in the paper. The orbital motion of the electrode tool decoupled the size of the hole to be drilled from the size of the electrode tool, so enabling a range of hole sizes to be drilled. The orbiting motion can increase the hole diameter proportional to the orbit radius, which enhances flushing because the gap between the work piece and the electrode tool became larger. As regards large depth to diameter ratio holes, the increased flushing reduced electrode tool wear, created a better surface finish and eliminated the exponential reduction in material removal rates typical for EDM drilling. The study reported that holes drilled using the orbiting technique had very smooth and uniform surfaces. Figure 20 shows the scanning electron microscope image of the cross-sections at 350× magnification of drilling, with and without orbiting.

Similarly, Rajurkar and Shen [179] presented an approach based on the planetary movement of an electrode tool to create the extra space needed for debris removal from the narrow gap when drilling high aspect ratio micro-holes as well as blind non-circular micro-holes. This proposed approach was verified by drilling through micro-holes with an aspect ratio of 18 and blind non-circular micro-holes with sharp corners and edges. The process performance characteristics, such as the MRR and the electrode tool wear, were analysed for stainless steel AISI 304L under different machining conditions. Furthermore, Masuzawa, et al. [168] proposed a technique to operate the micro-EDM machine in a manner similar to a turning lathe. The electrode fabrication system was installed to allow the possibility of fabricating complex microelectrodes with sharp edges and corners on-the-machine. Based on set of equipment to test the machining of a real prototype, the authors demonstrated that the proposed process can produce micro-cylindrical, overhung cavities or cylindrical micro-holes with larger internal diameters than the entrance diameter. Lin and Lee [74] enhanced the EDM process with a strong magnetic force which can both assist in clearing debris and enhancing surface finish. Using the Grey relational analysis they showed that the production parameters could be optimized to obtain high MRR, low electrode tool wear and good surface quality.

Sharma, et al. [217] used rotary tubular copper and brass electrodes tool for EDM of AISI 329 stainless steel. It was revealed that a copper electrode tool performed better than a brass electrode tool in terms of hole quality, MRR and EWR. Muthuramalingam, et al. [218] used brass and tungsten carbide tools to investigate the effect of tool re-solidification on surface hardness during EDM of AISI 202 stainless steel. It was revealed that the surface hardness of the workpiece increased with the tungsten carbide electrode tool whereas it decreased with the brass electrode tool due to layer formation on the workpiece.

Gohil and Puri, [163] employed EDM for turning purpose, they designed a turning spindle to generate free-form cylindrical geometries on SS-304 stainless steel work piece. The study investigated the effect of machining parameters, such as pulse-on time, peak current, gap voltage and tool thickness on the MRR and TWR. Alshemary, et al. [208] used different experimental parameters such as, pulse on time, pulse off time and wire tension to study the types of errors generated on the feature machined by the wire EDM of 2205 duplex stainless steel. Also, the study explored the effect of these parameters on the on cylindricity error, circularity error and diameter error. The author reported that, Wire tension has highest contribution on cylindricity error which is lowest at high value wire tension. Pulse on time has minor contribution on the cylindricity error and it increases with the increase of pulse on time. Pulse of time does not have any influence on the cylindricity error. The circularity error was lowest at medium pulse off time and medium wire tension; and those two parameters have almost similar and highest contributions. The pulse on time has around 14% contribution on circularity error and the medium value of it minimizes the circularity error. The input parameters such has pulse on time, pulse off time and wire tension have around 13%, 16% and 7% contributions respectively on diameter error which is minimized at medium pulse on time and low pulse off time and low wire tension.

### 4.3. Layer Machining Research of Stainless Steel

Stainless steel materials were also considered in the study of layer machining where complex structures are built up layer by layer using micro-EDM. Li, et al. [167] proposed a compensation method based on the scanned area (BSA) of each layer. The 3D micro-cavities were generated by integrating the EDM with a computer aided design and computer aided manufacturing (CAD/CAM) system. The authors reported the experimental results of machining stainless steel AISI 304 in 3D using the proposed method and compared the results with those obtained using the uniform wear method and those using a combination of linear compensation with uniform wear method. The proposed method was an integration of the electrode tool wear compensation method with the CAD/CAM system. It was claimed that with the proposed method machining efficiency was improved and the tool wear ratio was reduced.

**Figure 21:**
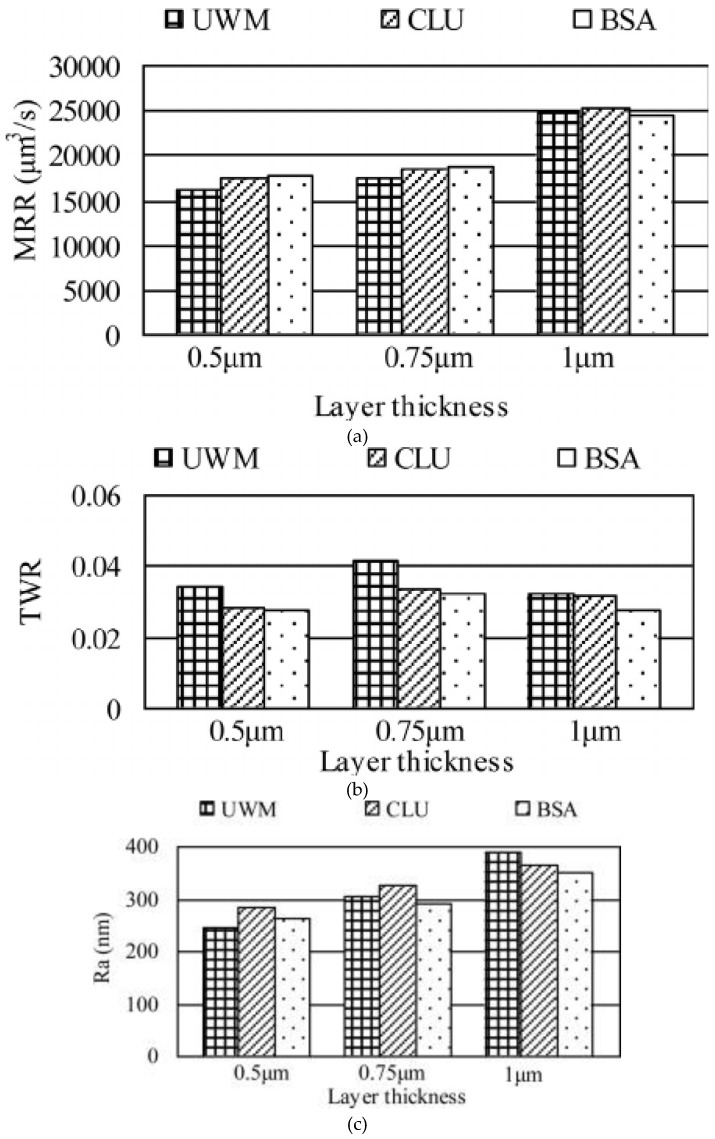
(**a**) MRRs for the three methods UWM. CLU and BSA; (**b**) TWRs for the three methods; and (**c**) SR for the three methods [167].

Figure 21a–c show a comparison of the MRRs, TWRs and SR for the three methods namely uniform wear method (UWM), combination of linear compensation with uniform wear method (CLU) and BSA with 0.5 µm, 0.75 µm and 1 µm layer thicknesses, respectively. Yu, et al. [219] proposed a uniform wear method for the 3D micro-EDM with round or rectangular section electrode tools developed for micro-moulds. In this compensation method, the uniform wear at the end of the electrode tool was realized by layer-by-layer machining. The authors reported that complicated 3D cavities were successfully machined by combining linear electrode tool wear with uniform longitudinal compensation, thus effectively converting 3D electrode tool wear to a linear process.

Muthuramalingam [189] studied the influence of the pulse generator systems on white layer formation in EDM process. Based on experiment results, the author reported that, the iso energy pulse generator has created thinner and more symmetrical white layer thickness on the machined AISI 202 stainless steel workpiece owing to its ability to produce lower discharge energy pulses with nearly. Peak current was the significant factor on determining white layer formation in the EDM process.

### 4.4. Combined and Hybrid Processes for Stainless Steel

**Figure 22:**
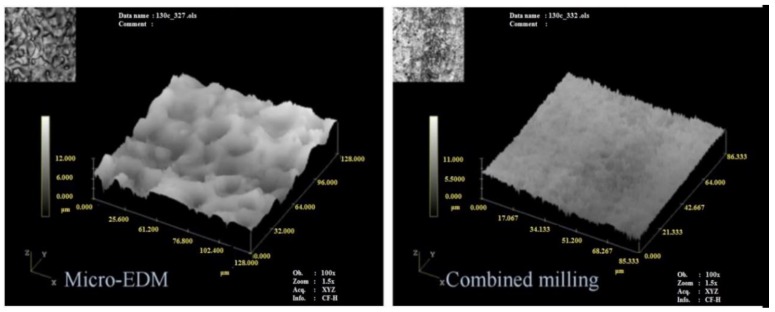
SR of the bottom surface [171].

**Figure 23:**
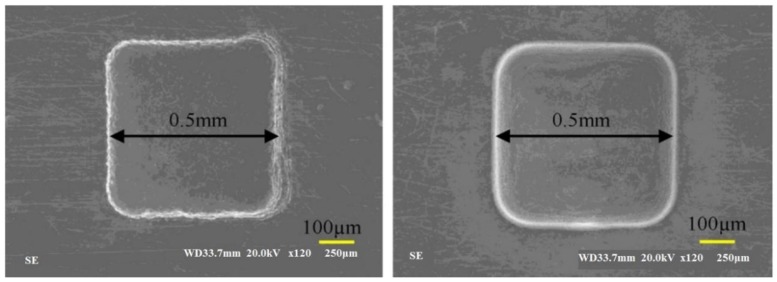
Scanning electron microscope (SEM) photos of the square cavity machined by EDM and micro-EDM combined with milling [171].

Many studies introduced hybrid process for EDM [220]. Stainless steel materials were also considered in many hybrid processes. Zeng, et al. [171] investigated the combining of micro-EDM and micro-electro-chemical machining (ECM) for the milling of a 3D micro-structure. The micro-EDM shaping and micro-ECM finishing were performed in sequence on the same machine tool with the same electrode tool but different dielectric media. The processing conditions were experimentally investigated for 304 stainless steel. The electrode tool used in both processes was fabricated online using an anti-copying block. The authors reported that the machining precision and shape accuracy were much better than parts machined using only micro-ECM milling. They also found that the SQ and mechanical properties of the workpiece were improved. The study demonstrated that this combined milling method was possible and useful in the field of 3D metallic micro-structure milling. Figure 22 shows the scanning electron microscopy (SEM) morphologies of the SR of the bottom surface. Figure 23 shows the SEM photos for squire cavity shape machined using only EDM compared to those machined by combined milling with machining gap of 10 µm with different gaps.

**Figure 24:**
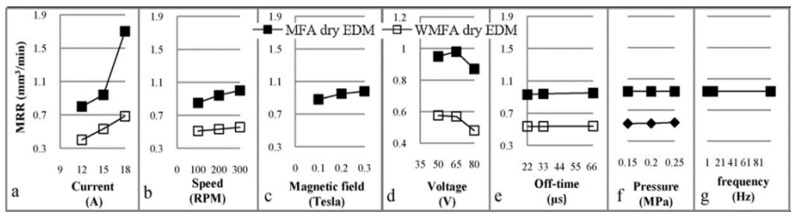
Comparative parametric plots for the MRR in MFA dry EDM and WMFA dry EDM [136].

**Figure 25:**
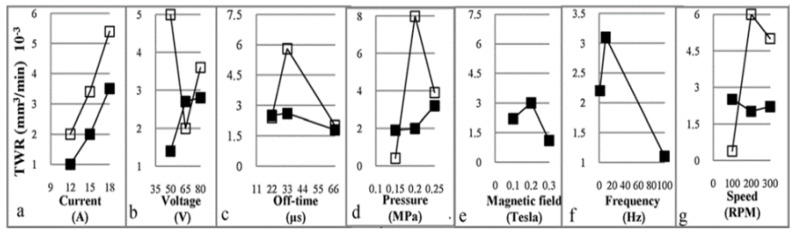
Comparative parametric plots for the TWR in MFA dry EDM and WMFA dry EDM [136].

Govindan, et al. [136] presented another hybrid process when they assessed material removal in pulsed magnetic field-assisted dry EDM. The application of the pulsating magnetic field increased the movement of electrons and the degree of ionization in the plasma. Based on experiments of two stainless steel split workpieces with parametric variations, the authors reported that this hybrid approach led to productivity improvement of 130%, with zero tool wear, as compared to the dry EDM process without the magnetic field. An improvement in the SQ was also found by the SEM. Figure 24 and Figure 25 show a comparison between the MRR and the TWR in the dry EDM process performance with the magnetic field architecture (MFA) and without magnetic field (WMFA).

Aligiri, et al. [194] proposed a combination of the single electrical discharge electro-thermal model with online monitoring of the EDM inter-electrode gap to estimate the material removal volume in real time. An electro-thermal model was used to estimate unit material removal volume, while the online monitoring process was employed to count the number of discharge pulses and discriminate the micro-EDM pulse. The pulse distribution was used to illustrate the ongoing inter-electrode gap condition. A new micro-EDM drilling scheme was developed by utilizing the real-time estimator of the material removal volume, which enabled compensation for the effect of tool wear and led to the fabrication of accurate micro-holes. The authors reported that the experimental and estimated results were found to be in satisfactory agreement with the average error less than 14.3% for stainless steel, titanium and nickel alloy workpieces under various energy input and machining depth settings.

Yu, et al. [221] combined ultrasonic vibration with the planetary movement of an electrode tool to drill micro-holes with high aspect ratios using micro-EDM. Stainless steel was used to experimentally evaluate the proposed method. The authors reported that the wetting effect of the ultrasonic vibration was larger than the effect of the uneven distributed gap provided by the planetary movement of the electrode tool. Another combination of the ultrasonic and electrical discharge machining has been presented by Gao and Liu [41], where the proposed method was to vibrate the workpiece during machining. Based on the experimental results of machining stainless steel and copper, the authors reported that the induced workpiece vibration significantly affected the performance of the micro-EDM. Furthermore, the efficiency and the aspect ratio of the hole of the ultrasonic-aided micro-EDM noticeably increased. Chen, et al. [156] designed a mechanism of cutting a pipe using EDM to avoid the pipe deformation, residual stresses and loss of strength usually obtained with traditional pipe cutting mechanisms. The mechanism was set up on the table of the EDM machine. This combination was utilized to study its effect on the machining parameters, such as the MRR and relative TWR and on machining characteristics. The machining variables, including machining polarity, peak current, pulse duration and electrode tool rotary speed, were chosen to explore the machining efficiency. Curodeau, et al. [222] presented a hybrid EDM process that used a polymer-carbon electrode tool that could be re-moulded repeatedly into complex geometries and used to perform precision EDM machining in de-ionised water. It was found this method improved the SR.

Rajurkar and Yu [169] proposed an approach to integrate CAD/CAM systems into micro-EDM to compensate for tool wear using a uniform wear method. The authors verified the approach by successfully generating very complex 3D micro-cavities in stainless steel AISI 304. In addition, the feasibility of the approach was demonstrated by generating complex macro-cavities using conventional EDM with single, simple-shaped electrode tools. Wang, et al. [223], suggested a novel super high speed EDM milling and arc machining process. Compared with conventional EDM, the hybrid process performed exceptionally well in terms of MRR, of nearly 21,500 mm^3^/min. while the EWR remained similar to the original process.

### 4.5. Effect of the EDM Process on the Properties of Stainless Steel and the Machined Surface

**Figure 26:**
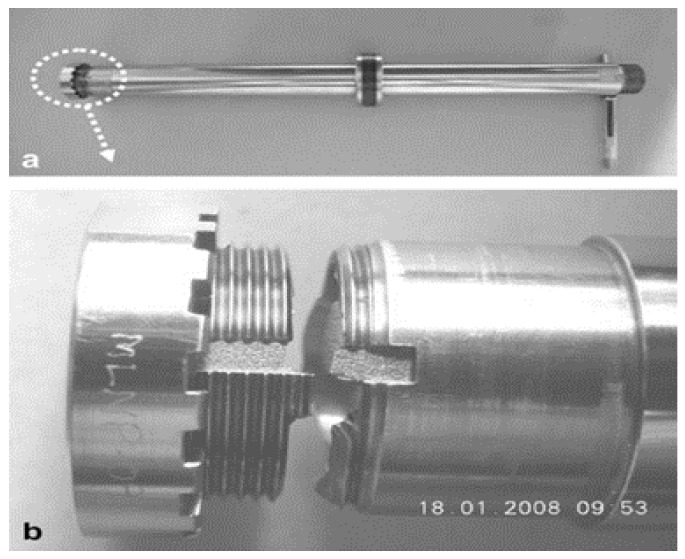
Photographs showing (**a**) the failed piston and (**b**) the location of failure [195].

The effect of the EDM process on the mechanical behaviour of stainless steel has been studied by many researchers such as [224]. Jha, et al. [195] studied the role played by the EDM process in the fatigue performance of 15–5 PH stainless steel. The study conducted qualification tests of a piston workpiece made of the given stainless steel. The piston had fractured under cyclic loading with the fracture initiated at the surface machined by EDM and propagated under the cyclic loading (Figure 26).

Huang, et al. [191] have presented a microstructure analysis of a martensitic stainless steel, AISI 440A, surface fine-cut by WEDM. In this study, the surface of the stainless steel workpiece had been tempered at 600 °C and was finished by multi-cutting by WEDM. The study utilized scanning and transmission electron microscopes integrated with an energy-dispersive X-ray spectrometer to study the machined surface microstructure. The authors reported that a heat-affected zone (HAZ) of about 1.5 µm thick was developed in the surfaces machined with a negatively polarized wire electrode. A few spherical deposits of wire material covered with oxides with circa 10 nm thickness were also registered. The authors reported that no obvious HAZ was detected for the surface finished with a positively polarized wire electrode. A very thin uniform layer (<50 nm) composed of wire electrode and workpiece material was found.

**Figure 27:**
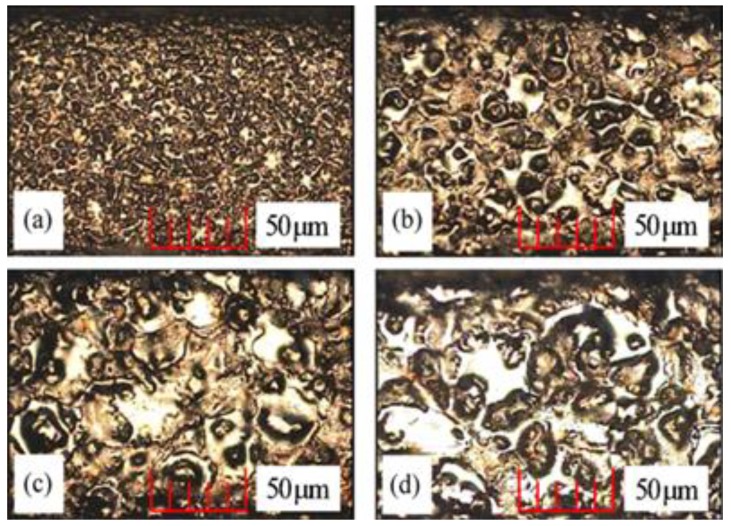
The surface roughness caused by various peak currents: (**a**) 1.5 A (0.715 µm), (**b**) 3 A (0.796 µm), (**c**) 5 A (0.855 µm) and (**d**) 6 A (0.994 µm) [187].

Hung, et al. [187] explored the feasibility of using a relatively rapid technique, die-sinking micro-EDM, to fabricate miniature SUS316L bipolar plates. The study presented the relationships between MRR, discharge current and SR. The authors reported that the proposed technique accelerated the processing time with MRR increasing to 7.2 mm^3^·min^−1^. The high processing time led to an increase in SR. The maximum power density for smoother surfaces (0.715 µm) was 673.5 mW·cm^−2^, whereas that for the coarser surface (0.994 µm) was 646.2 mW·cm^−2^. Figure 27 shows the SR was generated by various peak discharge currents. The authors reported that the effect of the SR on cell performance was not obvious in their experiment.

Bhaumik and Maity, [161] studied the effect of deep cryogenically treated post tempered electrode tools during EDM operation of AISI 304 stainless steel. They considered process parameters including pulse on time, duty cycle, peak current, gap voltage and flushing pressure to investigate the process performance by means of radial overcut. Microstructural analysis has been carried out for the machined surfaces. The authors reported that, deep cryotreated post tempered electrode tools considerably decrease the radial overcut. Yu, et al. [173] developed a micro punching system with a micro electrical discharge machining (EDM) module. They reported that, the key dimensional error of blanked part varies by different materials, which is less than 2.5 μm. Cheong, et al. [207] studied the effect of connecting a MOSFET to a normal RC type pulse generator and using deionized water with low resistivity on the tool wear was reduced and geometrical accuracy. The authors reported that, by connecting a MOSFET to a normal RC type pulse generator tool wear was reduced and geometrical accuracy was increased during machining of stainless steel using cemented carbide tool. Using deionized water with low resistivity resulted in further reduction in tool wear and also reduce geometrical accuracy. The combination of using a MOSFET with low resistivity water therefore reduces tool wear while maintaining geometrical accuracy. The addition of the MOSFET to the RC circuit reduced the relative wear ratio by up to 67%.

Ho, et al. [201] proposed an experimental investigation of thermal strain caused by electrical discharge machining on a stainless-steel (SUS430) plate using digital image correlation. The authors reported that, the peak strain presented in the outer ring and the strain decreased inward toward the inner core. Experimental results revealed that the maximum thermal strain caused by electrical discharge machining was proportional to the drilling depth.

### 4.6. Dielectric Fluid Research

**Figure 28:**
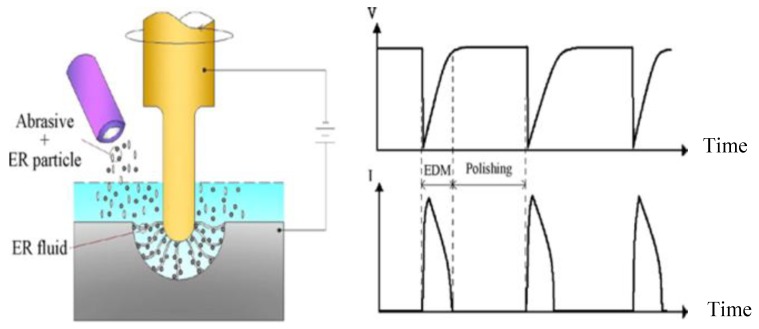
Schematic diagram of the proposed method using the ER fluid and abrasive grit [88].

**Figure 29:**
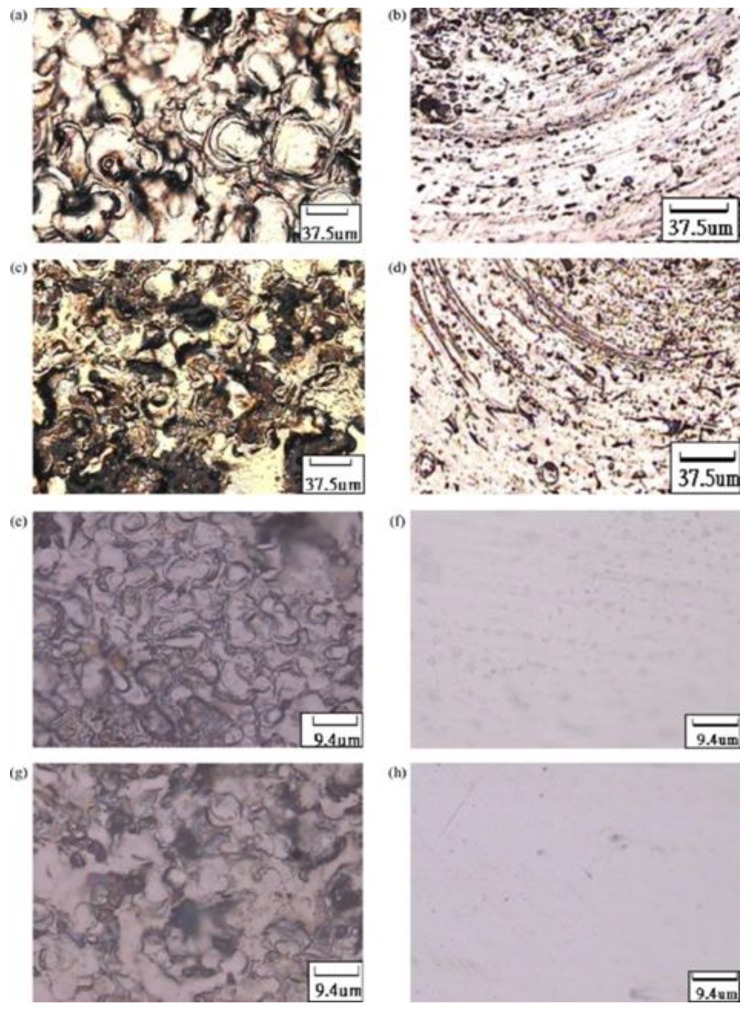
The machined surface for different concentrations of starch and abrasive Al_2_O_3_, under discharge capacitances of 0.068 µF and 0.01 µF: (**a**) 10 wt.% starch without Al_2_O_3_, SR = 1.24 µm C = 0.068 µF; (**b**) 10 wt.% starch - 10 wt.% Al_2_O_3_, SR = 0.26 µm C = 0.068 µF; (**c**) 20 wt.% starch without Al_2_O_3_, SR = 1.12 µm C = 0.068 µF; (**d**) 20 wt.% starch-10 wt.% Al_2_O_3_, SR = 0.14 µm C = 0.068 µF; (**e**) 10 wt.% starch without Al_2_O_3_, SR = 0.52 µm C = 0.01 µF; (**f**) 10 wt.% starch-10 wt.% Al_2_O_3_, SR = 0.06 µm C = 0.01 µF; (**g**) 20 wt.% starch without Al_2_O_3_, SR = 0.46 µm C = 0.01 µF; and (**h**) 20 wt.% starch–10 wt.% Al_2_O_3_, SR = 0.08 µm C = 0.01 µF [88].

Stainless steel materials have also been used in the study of the role of the dielectric fluid in EDM processes. Tsai, et al. [88] proposed a novel method that combined EDM and polishing into a single stage. Instead of a conventional EDM dielectric fluid such as de-ionized water and oil this method used an electro-rheological (ER) fluid to which fine abrasive grit was added. Figure 28 shows a schematic diagram of the proposed method. The experimental results for stainless steel (SUS304) demonstrated that the EDM process can still occur in the ER fluid and the polishing process is performed after adding the alumina abrasive. The authors reported that the roughness for a discharge capacitance of 0.01 µF was improved to 0.06 µm when using ER fluid with alumina powder with particles of 0.3 µm mean diameter. Figure 29 shows the machined surface using the ER fluid and the abrasive Al_2_O_3_ under different discharge capacitances.

Aghdeab, et al. [186] investigated the influence of using different dielectric such as vegetable oil, transformer oil and gas oil on MRR, surface roughness and whit layer thickness in EDM for stainless steel 316L specimens with a copper electrode tool. The authors reported that, the vegetable oil gives extreme MRR while the relative SR is lower and higher whit layer thickness when compared to transformer gas oils. Also, they reported that, the current and pulse-on-time are the most important factors that effect on MRR, SR and WLT. The MRR in vegetable oil and transformer oil produced 66% and 61% higher MRR, respectively, than gas oil. Additionally, vegetable oil and transformer oil resulted in 38% and 13% lower SR than gas oil, while vegetable oil and transformer oil resulted 28% and 14% higher in WLT than gas oil.

### 4.7. Other Research on Stainless Steel and EDM

Other studies have concentrated on the required energy of the discharge pulse. Mahardika, et al. [203] presented a fundamental study on the total discharge pulse energy needed to machine different operations. They proposed a λ, θ, ρ theory, where λ was the thermal conductivity; θ was the melting point; and ρ was the electrical resistivity of the workpiece. They introduced parameters that were independent of machining complications to measure the ease of machining. These parameters were the total energy of the discharge pulses, discharge pulse number, average discharge pulse energy, discharge pulse density and electrode tool wear. Based on the experimental results of machining stainless steel and other materials, these authors reported that the coefficient of correlation for each parameter from the λ, θ, ρ theory was much higher than for the λ, θ theory. The authors concluded that the λ, θ, ρ theory is better than the λ, θ theory for determining the ease of machining with the EDM process.

**Figure 30:**
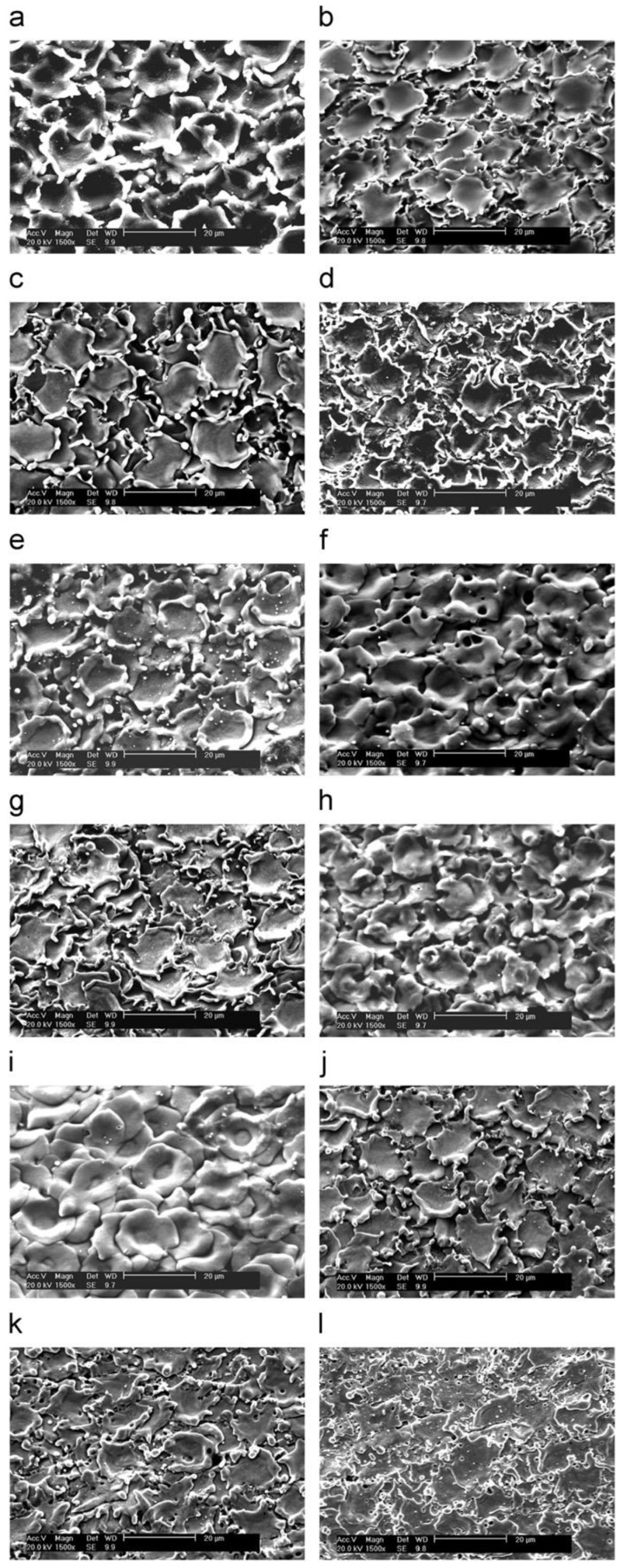
SEM images of the discharge craters for different workpiece materials: (**a**) aluminium (Al), (**b**) silver (Ag), (**c**) brass (Br), (**d**) copper (Cu), (**e**) nickel (Ni), (**f**) steel (Fe), (**g**) platinum (Pt), (**h**) titanium (Ti), (**i**) stainless steel, (**j**) tantalum (Ta), (**k**) molybdenum (Mo) and (**l**) tungsten (W) [203].

The study compared the ease of machining 12 workpiece materials using EDM; it was found that Aluminium was the easiest material to machine by EDM and Tungsten was most difficult, according to the λ, θ, ρ theory. The machining time of Al at 42.9 s was longer than the 37.4 s for W. The discharge craters for the 12 materials are shown in Figure 30.

Salah, et al. [183] carried out numerical modelling of the temperature distribution caused by the electric discharge machining process. The MRR and the total roughness were deduced from these results and compared with experimental observations for stainless steel type AISI 316L. Other researchers, such as Gao, et al. [41] have established a vibration model for the workpiece or for the tool (wire). Srivastava and Pandey, [216] concentrated on the kerf analysis in micro-WEDM machining and have determined the wire lateral vibration and the breakdown distance for the micro-WEDM process. They also advanced a wire lateral vibration model, which they used to calculate the maximum amplitude of wire vibration. These authors also performed numerous experiments on stainless steel with different machining parameters.

Simulation models have been developed in other studies. Zhang, et al. [197] used a fix-length compensation method to develop a 2D geometric simulation model for the micro-EDM milling process. The developed simulation model was used to predict the cone angle with a significant effect on the accuracy of the 3D micro-cavity. Based on the experimental results applied on a 1Cr18Ni9Ti stainless steel workpiece, the authors reported that the relative error of the simulation compared to the experimental data was within 4% under most machining conditions. Navas, et al. [225] made a comparison between EDM, grinding and hard turning processes with respect to residual stresses and surface integrity. The results showed that hard turning generates small tensile stresses in the surface, accompanied by an increase in hardness. Among the three processes, EDM generates least stresses but it was detrimental for surface integrity.

**Figure 31:**
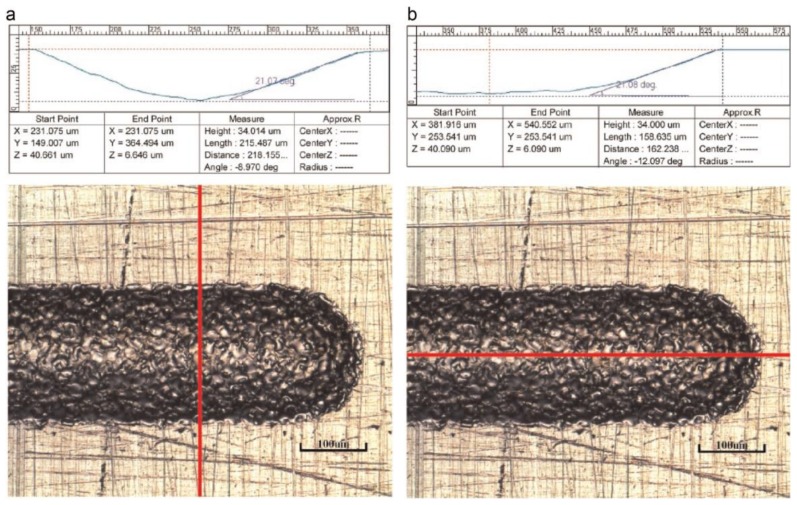
Actual machined results for the workpiece under Zeiss confocal microscope when the layer thickness is 43.5 μm: (**a**) cross section and (**b**) longitude section [197].

Etemadi, et al. [226] studied crack initiation in 17-4 PH stainless steel actuators made by EDM. The SEM results and hardness testing revealed that failure occurs due to fatigue because of the hardened layer produced by the EDM process. Post-machining polishing by means of fluidized bed granules solved this problem. Simulation models were developed in other studies. For example, Zhang, et al. [197] have utilized a fix-length compensation method to develop a two-dimensional geometric simulation model for micro-EDM milling process. The developed simulation was used to predict the cone angle which has significant effect on the accuracy of 3D micro cavity. Based on experimental results applied on the 1Cr18Ni9Ti stainless steel workpiece the author reported that the relative error of the simulation compared to the experimental data is within 4% under most machining conditions. Figure 31 shows the actual machined results for the workpiece.

## 5. Discussion

Reviewing the publications related to the stainless steel machining using the EDM process shows that the majority of studies investigated the effect of working parameters on performance parameters; mainly the MRR, EWR and SQ. Other research have been conducted to solve or study other issues, such as electrode tool shape and movement, effect of EDM process on the properties of stainless steel, the machined surface, combined and hybrid processes and the dielectric fluid used in the process, amongst others. Researchers have paid more attention to the die-sinking EDM and micro-EDM processes to identify optimal and near-optimal working parameters, an emphasis which may be attributed to the popularity of the two processes. In contrast, little or no attention has been paid to the powder-mixed EDM process of stainless steel machining.

**Figure 32:**
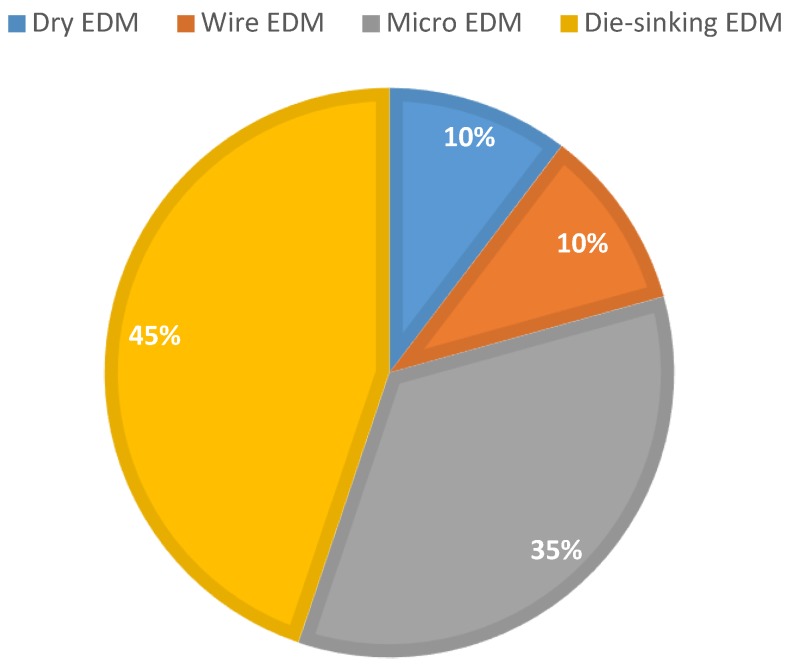
Relative usage of the different EDM processes utilized for studying stainless steel machining.

Most researchers have focused on parameter optimization rather than process innovation. Hybrid processes are now becoming a hot research topic but this is a new area and will require much work before it can be successfully adopted by industry. Micro-EDM is also becoming prominent due to the demands by industry for miniaturization. Electrical discharge turning (EDT) is an upcoming field now emerging as a domain within EDM [227,228]. Figure 32 shows the relative usage of different EDM processes used for stainless steel machining. Table 4 shows a summary of the studies on the EDM processing of stainless steel.

## 6. Conclusions

This review on the state-of-the-art studies on the EDM processing of stainless steels leads to the following conclusions:

- A number of research studies have been carried out in the field of EDM and significant improvements in the properties of the machined surfaces have been reported.
- Despite promising results, EDM processes for new materials used in industry still have problems, including low MRR and high TWR. These issues require further investigation.
- The reported results generally agree, for machining different stainless steel grades on the EDM the main factor influencing the MRR is the discharge current. However, pulse duration time, gas pressure and electrode tool rotation speed also have a significant influence on the MRR and the MRR can be further improved by using strip-EDM instead of wire-EDM.
- The review reveals that the main parameters influencing the EWR are the discharge current and pulse duration time.
- The reports generally agree that the SR decreases and better surface finish was achieved, with lower values of pulsed current and pulse-on time and relatively higher pulse-off time. High quality SR cannot be achieved where long-duration pulses are used during the finish machining.
- The review reveals that the crack length is significantly influenced by voltage, current, pulse off-time and speed in the wall and bottom regions of holes machined using dry EDM.
- The review also reveals that workpiece vibration caused by ultrasonic action can improve the performance and efficiency of the micro-EDM process by many times compared to the EDM processes without ultrasonic vibration.
- Applying a magnetic field can improve the geometric and surface quality in the dry EDM process. Using a magnetic field can also lead to a higher transfer of thermal energy to the workpiece and improves the removal of melted material with the dry EDM.
- The orbiting technique provides more uniform geometries of the machined hole and greatly improves the bottom quality for blind holes. The technique also reduces the tooling needs and the electrode tool wear but increases machining times.
- Most researchers paid attention to parameter optimization rather than process improvement or innovation.
- A general observation by the researchers was that the use of electro-rheological fluid can improve SR. Further improvements in the SR can be achieved if electro-rheological fluid is mixed with abrasive powder. There are few existing studies on this topic and more studies are needed to investigate the effect of adding different powders to the EDM of stainless steel.
- Much deeper and more accurate micro-holes can be machined and lower tool wear ratios can be achieved using a high spindle speed.

## 7. Future Research Directions

Despite so much work in the field of EDM, there remain issues which require further investigation, these are listed as follows:

- *Optimizing Process Parameters.* The EDM process has a very complex nature due to the complicated discharge mechanisms and their interactions and this makes it difficult to globally optimize the process. The advent of new materials always makes parameter optimization a hot research area and as new grades of stainless steel are introduced frequently, so more investigations are required.
- *Extending EDM to a wider range of workpiece materials.* EDM is used mainly for conductive materials, however, there is a current trend to explore the possibilities of machining non-conductive or semi-conductive materials such as ceramics, using EDM.
- *Use of different electrode tools.* Researchers can investigate the performance of the EDM process by using electrode tools of different materials, shape, size and geometry. Use of tubular electrode tools is in its infancy but has already provided promising results, it requires further attention.
- *Electrode tool cooling methods.* Electrode tool cooling is another research field of potential benefit. Cryogenic cooling of electrode tool has provided positive results in terms of reduction in TWR.
- *Hybrid or assisted EDM.* The EDM process hybridized with other processes can provide better results than EDM on its own. Magnetic force assisted EDM, laser assisted EDM and so forth, have the potential to overcome many process limitations. For example, ultrasonic assisted EDM showed significant improvement in performance. Research trends may be directed toward the combination of several processes.
- *Powder mix EDM.* Powders of different materials mixed with dielectrics have been shown to improve the machining process. This is another area which requires further attention and researchers need to address the machining of different stainless steel grades using different EDM processes with dielectric fluids containing different material powders.

**Figure 33:**
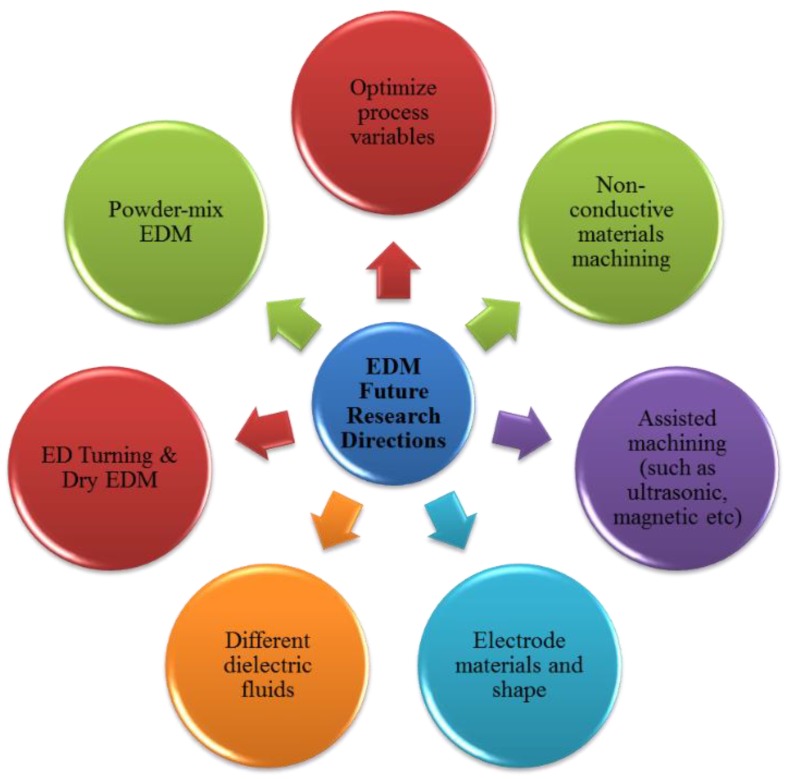
Possible future research areas in EDM.

Figure 33 shows a pictorial representation of possible future research directions.

**Figure 34:**
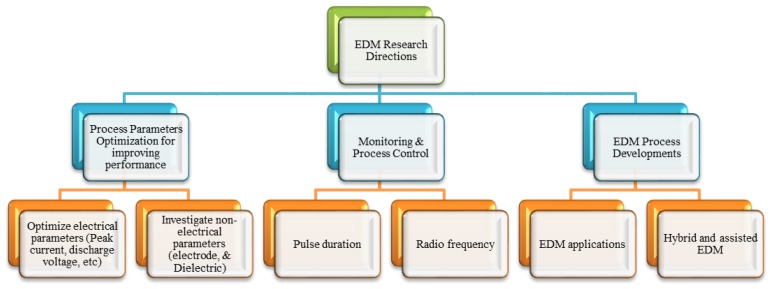
Classification of possible future research directions.

Future research directions can be classified into four broad categories which can be further divided into sub-categories as shown in Figure 34.

## Figures and Tables

**Table 1:** Designations, compositions, mechanical properties and typical applications for austenitic, ferritic, martensitic and precipitation-hardenable stainless steels [7].

AISI Number	UNS Number	Composition (wt.%) ^a^	Condition ^b^	Mechanical Properties			
				Tensile Strength [MPa (ksi)]	Yield Strength [MPa (ksi)]	Ductility [%EL in 50 mm (2in.)]	Typical Applications
**Ferritic**							
409	S40900	0.08 C, 11.0 Cr, 1.0 Mn, 0.50 Ni, 0.75 Ti	Annealed	380 (55)	205 (30)	20	Automotive exhaust components, tanks for agricultural sprays
446	S44600	0.20 C, 25 Cr, 1.5 Mn	Annealed	515 (75)	275 (40)	20	Valves (high temperature), glass moulds, combustion chambers
**Austenitic**							
304	S30400	0.08 C, 19 Cr, 2.0 Mn, 9 Ni	Annealed	515 (75)	205 (30)	40	Chemical and food processing equipment, cryogenic vessels
316L	S31603	0.03 C, 17 Cr, 2.0 Mn, 2.5 Mo, 12 Ni	Annealed	485 (70)	170 (25)	40	Welding construction
**Martensitic**							
410	S41000	0.15 C, 12.5 Cr, 1.0 Mn	Annealed Q&T	485 (70) 825 (120)	275 (40) 629 (90)	20 12	Rifle barrels, cutlery, jet engine parts
440A	S44002	0.70 C, 17 Cr, 1.0 Mn, 0.75 Mo	Annealed Q&T	725 (105) 1790 (260)	415 (60) 1650 (240)	20 5	Cutlery, bearings, surgical tools
**Precipitation Hardenable**							
17-7PH	S17700	1.0 Al, 0.09 C, 17 Cr, 1.0 Mn, 7 Ni	Precipitation hardened	1450 (210)	1310 (190)	1–6	Springs, knives, pressure vessels

^a^ The balance of the composition is iron; ^b^ Q & T denotes quenched and tempered.

**Table 2:** Details of EDM process research studies for different grades of stainless steels.

Grades and Corresponding Machining Operations	Composition (wt.%)	Properties
**AISI (SUS) 304** Die-sinking EDM [88,156,157,158,159,160,161,162,163,164,165] Wire EDM [166] Micro-EDM [167,168,169,170,171,172,173] Dry EDM [48,174,175,176,177] Powder-mixed EDM [88]	C ≤ 0.08, Cr 18.00–20.00, Mn ≤ 2.0, Ni 8–10.5, P ≤ 0.045, S ≤ 0.03, Si ≤ 1.00	Excellent corrosion resistance and very good drawability. It has low yield strength and high elongation. It can be welded by all fusion and resistance welding processes [178].
**AISI 304 L** Micro-EDM [179]	C ≤ 0.03, Cr 18.00–20.00 Mn ≤ 2. 00, Ni 10.00–13.00, P ≤ 0.045, S ≤ 0.030, Si ≤ 1.00,	The low carbon version of 304. It has good resistance to carbide precipitation and so is recommended for corrosion resistance in water [178].
**AISI (SUS) 316** Die-sinking EDM [180] Wire EDM [181] Micro-EDM [182]	C ≤ 0.08, Cr 16.00–18.00, Mo 2.00–3.00, Mn ≤ 2.00, Ni 10.00–14.00, P ≤ 0.045, S ≤ 0.030, Si ≤ 1.00,	It has excellent corrosion resistance. Subject to pitting and crevice corrosion in warm chloride environments and to stress corrosion cracking above about 60 °C. It cannot be hardened by thermal treatment. It has excellent weldability by all fusion methods.
**AISI 316L** Die-sinking EDM [183,184,185,186] Micro-EDM [187]	C ≤ 0.03, Cr 16.00–18.00, Mn ≤ 2.00, Mo 2.00–3.00, Ni 12.00–15.00, P ≤ 0.045, S ≤ 0.030, Si ≤ 1.00,	The low carbon version of 316. It is more resistant to carbide precipitation and has higher strength at elevated temperatures.
**AISI 202** Die-sinking EDM [56,188,189]	C ≤ 0.15, Cr 17.00–19.00, Mn 7.50–10.00, N ≤ 0.25, Ni 4.00–6.00, P ≤ 0.060, S ≤ 0.030, Si ≤ 0.75,	Excellent toughness at low temperatures. When machined produces long, gummy chips. The material can be welded by fusion and resistance methods but should not be joined using oxyacetylene welding. Forging below 1010 °C (1850 °F) is not advisable for this grade [190].
**AISI 440 A2** Wire EDM [108,191]	C 0.39, Cr 15.89, Mo 1.02, Mn 0.87, P < 0.003, S < 0.003, Si 0.46,	This grade has high hardness, wear resistance and strength. It loses mechanical properties by over-tempering; therefore, it should not be used at temperatures below the relevant tempering temperature. It is fully annealed at 850 to 900 °C.
**AISI 420** Wire EDM [192]	C 0.16–0.25, Cr 12.00–14.00 Mn ≤ 1.00, P ≤ 0.040, S ≤ 0.030, Si ≤ 1.00,	A high-carbon steel with minimum chromium content of 12%. It offers good ductility in its annealed state and excellent corrosion resistance properties when the metal is polished, surface ground or hardened. It has corrosion resistance.
**Modified AISI 420** Wire EDM [193]	C 0.38, Cr 13.6, Mn 0.5, Si 0.9, V 0.3	Similar to grade 420 with more carbon content.
**Ferralium 255 SD 50 (plate)** Micro EDM [194]	C max 0.03, Cr 24.50–25.50, Cu 1.5–2.0, Mn 0.8–1.2, Mo 3.20–3.80, N 0.21–0.24, Ni 5.60–6.50, P ≤ 0.035, S ≤ 0.030, Si ≤ 0.4	Super duplex ferralium 255 SD50 has high yield strength, withstanding stresses of over 550 N/mm^2^. It has excellent corrosion resistance to corrosive. In seawater it offers superior resistance to crevice corrosion and pitting. It shows excellent ductility and impact resistance combined with a great resistance to abrasion, erosion and cavitation erosion.
**19-5PH** Die-sinking EDM [195]	C ≤ 0.07, Cr 14.00–15.50, Cu 2.50–4.50, Mn ≤ 1.00, P ≤ 0.040, Nb + Ta 0.15–0.45, Ni 3.50–5.50, S ≤ 0.030, Si ≤ 1.00,	Exhibits high strength and hardness with moderate corrosion resistance; has high toughness, especially in the through-thickness (short transverse) direction. Used in applications that require high transverse strength and toughness [196].
**1Cr18Ni9Ti** Micro EDM [197]	C max 0.12, Cr 17.0–19.0, Mn ≤ 2.00, Ni 8.0–11.0, P 0.035, S 0.030, Si ≤ 1.00, Ti 5 × (C% 0.02−0.80)	Has a good wear resistance [198].
**Other grades** Die-sinking EDM [199,200,201,202,203] Micro-EDM [41,204,205,206,207] Dry EDM [136] Wire EDM [208,209]	Grade not mentioned	Grade not mentioned

**Table 3:** Summary of selected studies in optimizing the machining process parameters of stainless steel.

No.	Authors (Ref)	Process	Machining Process	Machining Performance	Remark (note)
1	[48]	Dry EDM	Polarity, current, pulse duration time, gas pressure and electrode tool rotation speed	MRR and REWR (Relative Electrode tool Wear Rate)	The MRR value of this method can be improved by 2 or 3 orders of magnitude compared to conventional methods. MRR ↑ as (discharge current, pulse duration time, gas pressure and electrode tool rotation speed) ↑. The maximum MRR occurred at pulse duration = 9 µs and pulse interval = 2 ms. REWR ↑ as (discharge current and pulse duration time)↑ and ↓ as gas pressure↑
2	[193]	Wire EDM	Pulse-on, pulse- off, current and bed speed	Accuracy, SR, volumetric MRR and EWR	Group method data handling technique provided better prediction than multiple regression analysis.
3	[188]	EDM	Voltage, current and duty factor	MRR, SR	The iso duration current pulse generator produced better surface quality and higher material removal rate compared to the transistor pulse train generator. Discharge current and duty factor have most influence on determining the machining performance in EDM.
4	[166]	Wire-EDM	Peak current, radial depth of cut	MRR, SR	(Machining speed and SR) ↑ as the peak current ↑. MRR ↓ as radial depth of cut ↑. The MRR of the strip-EDM turning was 74.3% higher than the MRR for wire-EDM turning.
5	[177]	Dry EDM	Flushing gases	MRR	The proposed method improved the removed material per spark based on the properties of the oxidized particles and also enhanced the flushing efficiency of the process.
6	[175]	Dry EDM	Voltage, current, pulse-off time, oxygen pressure, spindle speed and clearance	Surface cracks	Average crack length in the wall and bottom regions of a hole machined by dry EDM was significantly influenced by voltage, current, pulse-off time and speed.
7	[56]	EDM	Current, pulse-on time and pulse-off Time	MRR and SR	The main factor influencing the MRR was the discharge current.
8	[176]	Dry EDM	Electrode tool and work piece material	MRR	The breakdown mechanism in the gas filled work gap was different from that experienced in traditional EDM, where the gap is filled with liquid dielectric.
9	[160]	EDM	Spindle speed	MRR and accuracy	Much deeper and more accurate micro-holes can be machined and lower tool wear ratio can be achieved under high spindle speed
10	[157]	EDM	Pulse-on time, pulse-off time, voltage and current	MRR	Current and pulse time were the most influential factors on the MRR.
11	[136]	Dry EDM	Voltage, current, pulse-off time, oxygen pressure, electrode tool speed, magnetic field and switching frequency	MRR, TWR and surface topography	Using a magnetic field led to higher transfer of thermal energy to the workpiece and improved material removal in dry EDM. Applying the magnetic field also improved the geometric and surface quality.
12	[174]	Dry EDM	Voltage, current, pulse-off time, oxygen pressure, electrode tool speed and shielding clearance	MRR, TWR, oversize and SR	MRR was significantly affected by gap voltage, discharge current and electrode tool rotational speed. Optimal processing parameters to achieve maximum MRR and depth were (50 V, 18 A, 22 ms, 0.25 MPa, 300 rpm and 4.5 mm) and zero TWR was observed. The crater radius and MRR in the dry EDM were more than those in the liquid dielectric EDM at low input energies. At higher discharge energies, larger crater radius and MRR were observed.
13	[204]	Micro-WEDM	Open voltage, discharge capacitor, charge resistance, feeding speed, reference voltage and wire tension	Kerf width	The open voltage was the main factor influencing the kerf width in the micro-WEDM.
14	[182]	Micro-EDM	Orbit radius and capacitance	Machining time and MRR	The orbiting technique provided more uniform geometries of machined holes and greatly improved the bottom quality for blind holes. It reduced tooling needs and electrode tool wear but increased machining times.
15	[88]	EDM	Dielectric fluid	SR	Discharge frequency and pulse number ↓with ↑ in the concentration of starch and alumina. More starch particles and alumina powder reduced the discharge efficiency. Using electro-rheological (ER) fluid and starch particles without abrasive Al_2_O_3_ improved the SR. Adding the abrasive to the ER fluid improved the SR. The roughness of SR (= 0.3 µm) obtained with ER fluid improved to 0.06 µm with the addition alumina powder.
16	[156]	EDM	Pulse duration, workpiece rotating speed, electrode tool polarity and current	MRR and relative EWR	The removal rate of material was proportional to the current. MRR for SUS 304 ↑ as pulse duration ↑ and the EWR started to ↓ when pulse duration reached 80 µs. The MRR of SUS 304 was larger for cathode discharge than anode discharge. The largest MRR and the lowest EWR appeared when the workpiece was rotated at 8 rpm. The developed triple-electrode machining system had a great effect in increasing the MRR and decreasing the ERR compared to the single-electrode system.
17	[170]	EDM	Arc intensity, dielectric medium and pulse width	Particle size	The yield of larger particles ↑ with ↑ arc current. The size distribution width ↑ as pulse width ↑. The yield of particles of all sizes was higher in kerosene than in water. The required range of particles sizes could be achieved with higher available current intensities and narrow pulse widths.
18	[158]	Micro EDM	Voltage, current and on/off time of the pulse	MRR and TWR	The main parameters that affected the MRR are voltage, current and pulse-on/off time. The voltage and current were proportional to the MRR. But only current was proportional to the TWR. Gap ↑ as voltage and current ↑ and it ↓ as length of pulse-on time ↑. Shorter pulse-on duration achieved accurate machining with a higher removal rate and a lower tool wear rate.
19	[183]	EDM	Current density	SR and TWR	Considering the temperature dependence of the conductivity was important in achieving accurate numerical results to provide better correlation with the experimental observations.
20	[108]	WEDM	Current, discharge duration, time between pulses, feeding speed, wire tension and flushing pressure	Surface morphologies	The significance of surface alloying was proportional to the passive current density. The presence of the secondary anodic peak was attributed to the dissolution of copper, the main element of the wire–electrode material, from the alloyed surface.
21	[191]	WEDM	Discharge duration, time between pulses, feeding speed and wire tension	Micro- structure of the finished surface	A HAZ of about 1.5 µm thick was found in the finished surfaces with the negatively polarized wire electrode. Fine equiaxed martensitic grains of about 200 nm were composed the HAZ. No HAZ was found with use of the positively polarized wire electrode.
22	[41]	Micro-EDM	Ultrasonic driving voltage, workpiece materials, machining method and workpiece thickness	MRR	The workpiece vibration caused by the ultrasonic action had a significant influence on the performance of the micro-EDM process. For workpiece of 0.5 mm thickness, the efficiency of the micro-EDM with ultrasonic action was eight times greater than the micro-EDM without ultrasonic activation.
23	[192]	Wire EDM	Pulse width, time between pulses, wire tension and feed speed	SR	The artificial neural networks model was better than the response surface methodology in predicting the SR and the cutting speed.
24	[212]	Micro EDM	Applied energy, HAZ and foil susceptibility to corrosion	Nozzle stability	As the energy input ↓ the quality of nozzles produced by MEDM ↑. Nozzle performance ↓ as carbon content and MEDM input energy ↑.
25	[214]	Wire-EDM	Pulse-on time, pulse- off time, current, no-load voltage, servo reference voltage, capacitor setting and servo speed setting	SR and machining speed	The method developed can improve the efficiency and effectiveness of the process whereby the optimal parameters are determined.
26	[164]	EDM	Pulse current, pulse on time and pulse off time	SR	The pulse current and pulse on time are the most significant machining parameters on the obtained surface roughness values.
27	[163]	EDM	Pulse-on time, peak current, gap voltage and tool thickness	MRR and TWR	The pulse on time is the most influencing factor that affects MRR and TWR. Voltage and tool thickness also identified as significant parameters, however, its effect is less than pulse-on time.
28	[208]	Wire-EDM	Pulse on time, pulse off time and wire tension	cylindricity error	Wire tension has highest contribution on cylindricity error which is lowest at high value wire tension. Pulse on time has minor contribution on the cylindricity error and it increases with the increase of pulse on time. Pulse of time does not have any influence on the cylindricity error. The circularity error was lowest at medium pulse off time and medium wire tension; and those two parameters have almost similar and highest contributions
29	[184]	EDM	Gap voltage and pulse on-time	SR	At low level of operating parameters, the surface irregularities such as micro-globules and micro-cracks by copper electrode tool is lesser than the surface irregularities by other electrode tool materials. At high levels of operating parameters, a denser distribution of surface irregularities due to high electrical discharge efficiency was observed.
30	[185]	EDM	Peak current, servo voltage, pulse on time, pulse off time and servo speed	TWR	Peak current is the most significant parameter to the TWR value
31	[172]	Micro EDM	Polarity	TWR	The direct polarity has significant in reducing the tool wear over the reverse polarity for the three electrode tools and the material removal rate is maximized with the direct polarity
32	[199,200]	EDM	Peak current, pulse on time, pulse off time and tool lift time	MRR and SR	Peak current, pulse on time and tool lift time have significantly affected the material removal rate and surface roughness.
33	[180]	EDM	Discharge current, pulse on time and duty cycle	MRR and SR	The proposed method maximize MRR and minimize SR
34	[181]	Wire EDM	Pulse on time, pulse off time, current and voltage	MRR	Pulse on time and current are greatly influence on the material removal rate.
35	[162]	EDM	Peak current, pulse duration and electrode diameter	SR	EDM parameters have a significant influence on machining characteristic such that surface roughness
36	[209]	Wire EDM	Peak current, pulse on time and wire feed	MMR and SR	The pulse on time is most significant parameter with percentage contribution about 87.29%
37	[165]	EDM	Current, pulse on time, voltage and inter electrode gap	MRR, TWR and SR	

**Table 4:** Summary of the studies on the EDM processing of stainless steel.

No	Authors (Ref)	Process	Workpiece Material	Objective Function
1	[197]	Micro-wire EDM	1Cr18Ni9Ti	Predicting the cone angle and its effect on the accuracy of the 3D micro-cavity
2	[48]	Dry EDM	AISI 304	Influence of the working parameters on the performance parameters
3	[193]	Wire EDM	Stavax (modified AISI 420)	Influence of the working parameters on the performance parameters
4	[188]	Die-sinking EDM	AISI 202	Surface quality and performance measures in EDM of stainless steel
5	[159]	Die-sinking EDM	Stainless steel #304	Proposing a strip electrode and guide system to overcome the electrode tool wear problem during the EDM process
6	[166]	Die-sinking EDM	Stainless steel #304	Proposing strip-EDM in the EDM-turning process to overcome the tool electrode tool wear problem. The paper also studies the influence of the working parameters on the performance parameters
7	[177]	Dry EDM	Stainless steel #304	Influence of the working parameters on the performance parameters
8	[205]	Micro-EDM	Stainless steel plate	Studied the surface quality of micro-holes
9	[167]	Micro-EDM	Stainless steel #304	Layer machining
10	[175]	Dry EDM	Stainless steel #304	Influence of the working parameters on the performance parameters
11	[171]	Micro-ECM	Stainless steel #304	Investigated micro-EDM and micro-ECM for combined milling of a 3D micro-structure
12	[202]	Die-sinking EDM	Stainless steel	Developed a pulse generator capable of shutting off harmful pulses
13	[56]	Die-sinking EDM	AISI 202	Influence of the working parameters on the performance parameters
14	[176]	Dry EDM	Stainless steel #304	Influence of the working parameters on the performance parameters
15	[160]	Die-sinking EDM	Stainless steel #304	Influence of the working parameters on the performance parameters
16	[157]	Die-sinking EDM	Stainless steel #304	Influence of the working parameters on the performance parameters
17	[187]	Micro-EDM	SUS316L	This study explored the feasibility of using a die-sinking micro-electrical discharge machining technique to fabricate miniature metallic bipolar plates
18	[136]	Dry EDM	Stainless steel	Presented an investigation of the hybrid dry EDM process performed in a pulsating magnetic field for improving process performance
19	[194]	Micro-EDM	Ferralium 255 SD 50	Combined single electrical discharge electro-thermal model with online monitoring of EDM discharge gap to estimate the material removal volume in real time
20	[174]	Dry EDM	Stainless steel #304	Influence of the working parameters on the performance parameters
21	[195]	Die-sinking EDM	15–5PH	Studied the role of EDM on fatigue performance
22	[204]	Micro-EDM	Stainless steel	Concentrated on kerf analysis in micro-WEDM
23	[221]	Micro-EDM	Stainless steel	Combined ultrasonic vibration with planetary movement of an electrode tool to drill micro holes
24	[182]	Micro-EDM	Stainless steel #316	Reported on electrode tool orbiting of micro-holes
25	[203]	Die-sinking EDM	Stainless steel	Presented a fundamental study of the total energy of discharge pulses required to machine different workpiece materials
26	[88]	Die-sinking EDM	Stainless steel #304	Proposed a method of EDM that used ER fluid instead of water or oil. The paper also studied the influence of the working parameters on the performance parameters
27	[156]	Die-sinking EDM	Stainless steel #304	Designed a new mechanism for pipe cutting combined with EDM. Influence of the working parameters on performance parameters. The influence of working parameters on performance parameters was also studied
28	[170]	Die-sinking EDM	Stainless steel #304	Influence of the working parameters on the performance parameters
29	[158]	Micro-EDM	Stainless steel #304	Influence of the working parameters on the performance parameters
30	[183]	Die-sinking EDM	AISI316L	Presents numerical results concerning the temperature distribution caused by the EDM process
31	[108]	Wire EDM	Martensitic stainless steel	Studied the surface alloying behaviour of martensitic stainless steel
32	[191]	Wire EDM	AISI 440A	Studied a microstructure analysis of the martensitic stainless steel
33	[41]	Micro-EDM	Stainless steel	Presented a combination method of ultrasonic and electrical-discharge machining
34	[168]	Micro-EDM	Stainless steel #304	Proposed a new type of micro-EDM machine, where the machine operated similarly to a turning lathe
35	[179]	Micro EDM	AISI 304L	Presented a new approach based on the principle of planetary movement of an electrode tool
36	[169]	Micro-EDM	Stainless steel #304	Proposed an approach to integrate CAD/CAM systems with micro-EDM
37	[213]	Die-sinking EDM	Stainless steel	Studied the influence of the working parameters on the performance parameters
38	[219]	Micro-EDM	Stainless steel	Proposed uniform wear method for 3D micro-EDM with round or rectangular section electrode tools developed for micro-moulds
39	[192]	Wire EDM	AISI 420	Developed modelling techniques for a wire EDM process
40	[212]	Micro EDM	Stainless steel	Studied the effects of MEDM on the hole properties
41	[214]	Wire EDM	Stainless steel #304	Utilized a feed-forward neural network to correlate working parameters on performance parameters
42	[164]	Die-sinking EDM	Stainless steel #304	Studied the effect of the pulse current, pulse on time and pulse off time on the surface roughness
43	[163]	Die-sinking EDM	Stainless steel #304	Investigated the effect of machining parameters and tool thickness on the MRR and TWR
44	[208]	Wire EDM	2205 duplex stainless steel	Studied the types of errors generated on the feature machined
45	[184]	Die-sinking EDM	Stainless steel 316L	Studied the effect of operating parameters on the surface roughness
46	[161]	Die-sinking EDM	Stainless steel #304	Studied the effect of deep cryogenically treated post tempered electrode tools
47	[185]	Die-sinking EDM	Stainless steel 316L	Investigated the influence of EDM parameters on electrode tools wear rate
48	[172]	Micro EDM	Stainless steel #304	Investigated the effect of polarity in tool wear and MRR
49	[199,200]	Die-sinking EDM	17-4 Precipitation Hardening Stainless Steel	Obtained the optimal process parameters of EDM
50	[173]	Micro EDM	Stainless steel #304	Developed a micro punching system with a micro electrical discharge machining
51	[201]	Die-sinking EDM	SUS430	Investigated the thermal strain caused by EDM
52	[180]	Die-sinking EDM	AISI 316 Stainless steel	Optimized the machining parameters
53	[181]	Wire EDM	Stainless steel 316	Optimized of wire EDM processes parameters
54	[162]	Die-sinking EDM	Stainless steel #304	Characterized the electric discharge machining
55	[209]	Wire EDM	Stainless steel 410	Studied the effect of process parameters on material removal rate and surface roughness
56	[189]	Die-sinking EDM	AISI 202	Studied the influence of the pulse generator systems on white layer formation
57	[165]	Die-sinking EDM	Stainless steel #304	Studied the effect of machining parameters on the material removal rate, tool wear rate and surface roughness
58	[186]	Die-sinking EDM	Stainless steel 316L	Investigate the influence of dielectric on material removal rate, surface roughness and whit layer thickness
